# Better Resolved Low Frequency Dispersions by the Apt Use of Kramers-Kronig Relations, Differential Operators, and All-In-1 Modeling

**DOI:** 10.3389/fchem.2016.00022

**Published:** 2016-05-11

**Authors:** J. van Turnhout

**Affiliations:** Department of Chemical Engineering, Sect. Organic Materials and Interfaces, Delft University of TechnologyDelft, Netherlands

**Keywords:** all-in-1modeling, electrode polarization, KK conversion frames, logarithmic derivatives and differences, matching Debye kernels, multivariate apart-together fitting, spectral resolution, symbolic differential operators

## Abstract

The dielectric spectra of colloidal systems often contain a typical low frequency dispersion, which usually remains unnoticed, because of the presence of strong conduction losses. The KK relations offer a means for converting ε′ into ε″ data. This allows us to calculate conduction free ε″ spectra in which the l.f. dispersion will show up undisturbed. This interconversion can be done on line with a moving frame of logarithmically spaced ε′ data. The coefficients of the conversion frames were obtained by kernel matching and by using symbolic differential operators. Logarithmic derivatives and differences of ε′ and ε″ provide another option for conduction free data analysis. These difference-based functions actually derived from approximations to the distribution function, have the additional advantage of improving the resolution power of dielectric studies. A high resolution is important because of the rich relaxation structure of colloidal suspensions. The development of all-in-1 modeling facilitates the conduction free and high resolution data analysis. This mathematical tool allows the apart-together fitting of multiple data and multiple model functions. It proved also useful to go around the KK conversion altogether. This was achieved by the combined approximating ε′ and ε″ data with a complex rational fractional power function. The all-in-1 minimization turned out to be also highly useful for the dielectric modeling of a suspension with the complex dipolar coefficient. It guarantees a secure correction for the electrode polarization, so that the modeling with the help of the differences ε′ and ε″ can zoom in on the genuine colloidal relaxations.

## Introduction

Dielectric spectroscopy is a powerful technique to study electrokinetic phenomena, it determines the frequency dependence of the real and imaginary part of the permittivity, ε′ and ε″. The measurements can span a very broad frequency range. In electrokinetic spectroscopic studies the low frequency part is quite relevant. At low frequencies the dissipative loss in ε″ caused by ohmic conduction becomes prominent, often to such an extent that it overshadows the genuine dispersion or pure relaxation losses of the colloidal particles. The unwanted ohmic loss might thus hamper the resolution of nearby relaxation processes seriously. So, in order to reach a high resolution the contribution of the ohmic conduction to ε″ should be eliminated. Conduction may also reduce the diagnostic selectivity of electrochemical sensors, so cleaning ε″ from conduction might be beneficial here as well.

**Figure 1:**
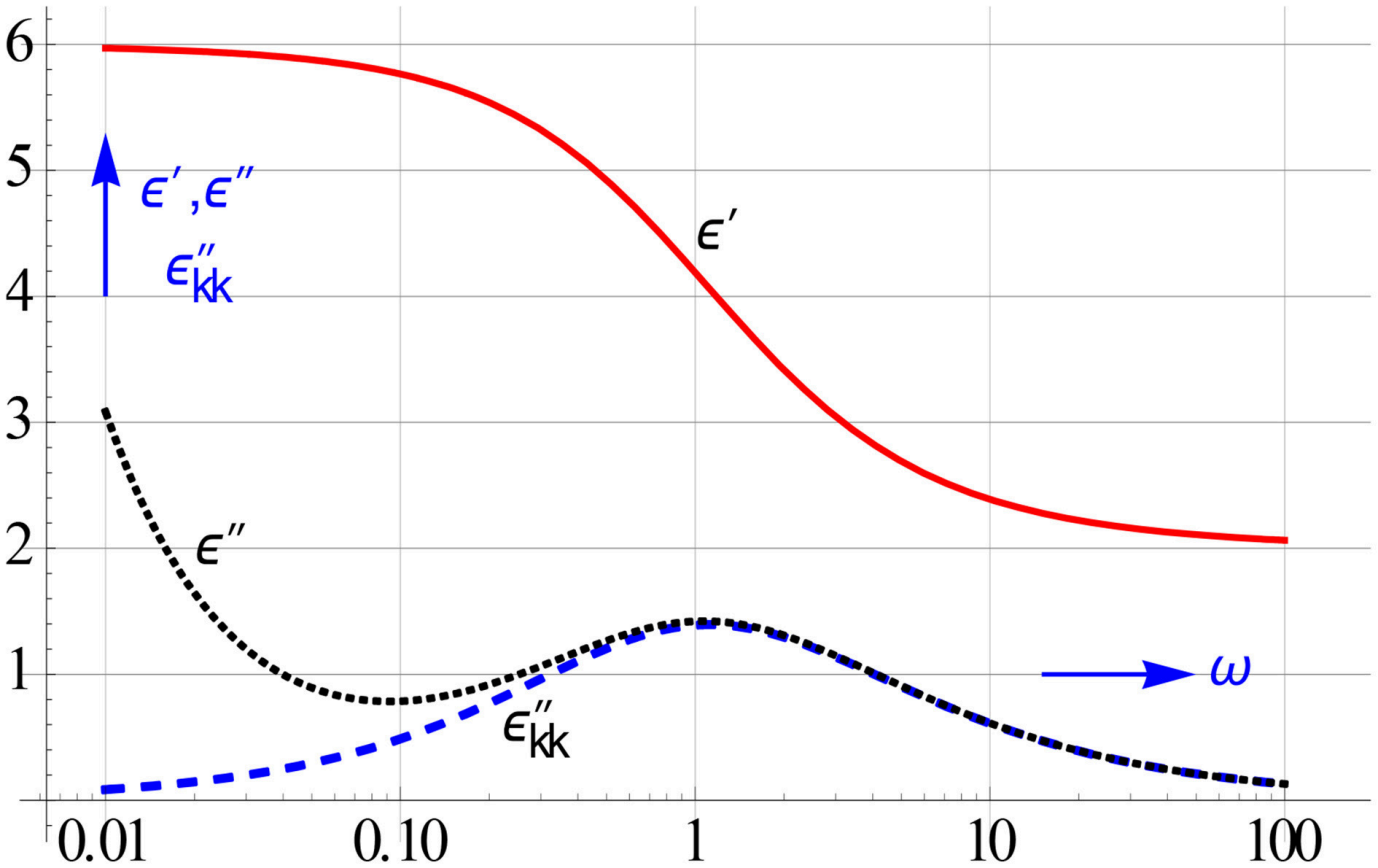
**Simulated observed ε″-spectrum and that of ε″ calculated by converting ε′-data into conduction free ε″-ones**. Only the latter shows us the authentic loss peak we look for. The ε′-spectrum remains unaffected by conduction. We assumed that the electrode polarization, which may also turn up at low frequencies, can be neglected, cf. **Figure 3**.

Figure 1 illustrates the problem caused by ohmic conduction. In order to reveal the hidden peak due to the true relaxation losses of the colloidal particles the ohmic conduction should be removed from the measured ε″-data.

**Figure 2:**
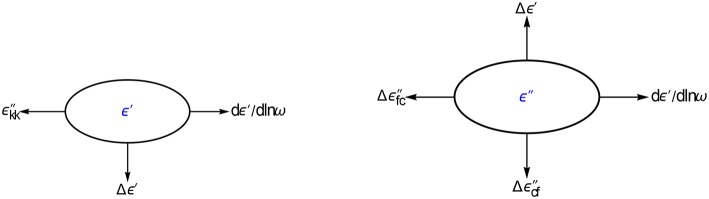
**Options explored for converting ε′ and ε″ data**. We will show that the differences of ε′ and ε″ offer new possibilities for improving the data analysis. All conversions are done with panels and executed as one-liners.

Clearly, the disturbing ohmic energy loss will not show up in the real part of the permittivity ε′, because ε′ is a measure of the number of dipoles and ions involved, but not of the energy required for their motion. Now one of the two Kramers-Kronig dispersion relations offers a means to calculate from the ε′ data, the genuine relaxation loss in the imaginary part ε″. Unfortunately such a KK interconversion is made difficult by the fact that the KK relations are singular integrals. We will describe two methods to approximate the unwieldy integrals accurately in an easy and fast way. This has the advantage that the ε′ to ε″ conversion can be coded as one-liners and can be used on line. The ε″ data obtained from ε′ will be denoted ε″_*kk*_ or ε″_*cf*_ (with cf being short for conduction free). The fast interconversion basically consists of a *frame* of coefficients by which ε″_*kk*_ can be calculated from a limited set of logarithmically spaced ε′ data clustered around the conversion frequency. Our compact KK relations in the form of moving conversion frames can in reverse be used to find the strength of the conduction loss from the difference of the observed and the converted ε″ data. This information is for example crucial for assessing the onset of percolation when the conducting phase in a colloidal mixture becomes co-continuous. A versatile way to avoid the problematic KK integrals is kernel matching. This mathematical tool relies on the fact that the permittivity can be considered to originate from a continuous distribution of elementary Debye relaxation processes. The distributed ε′ and ε″ do obey the KK relations but these improper integrals are no longer needed, because the conversion can now be accomplished by approximating the Debye kernel of the ε″ distribution-integral with a sum of logarithmic spaced Debye kernels of the ε′ distribution-integral. This singularity free approach thus provides the desired conversion panel with which ε″_*kk*_ can be uncovered readily by moving the panel along the observed logarithmically spaced ε′ data. The other method we have explored for the fast evaluation of the KK integrals is the approach of “integration by differentiation.” In this route the KK integrals are replaced by symbolic differential operators. It turned out that the logarithmic differential operators cannot be used in a broad sense. However, one operator, viz. the cot-operator scheme could be made useful for calculating *dε*′∕*dlnω* from a narrow window of logarithmically spaced ε″data. This logarithmic derivative can of course also be calculated from ε′ proper, in that case it will be automatically conduction free. The ‘loss’ peaks appearing in the *dε*′∕*dlnω* spectra have the advantage of being sharper than their corresponding ε″ counterparts, which implies that the resolution increases^1^. The various conversions we will discuss are summarized in Figure 2. In addition to $\varepsilon_{kk}^{\prime \prime }$ and *dε*′∕*dlnω*, we will also pay attention to the special features of the logarithmic differences of ε′ and ε″.

Another option to increase the resolution of lf-dispersions or actually of all dispersions is to compute the underlying distribution function by inverting the ε′ and/or ε″ data. For the latter we clearly should take its pure relaxation part, i.e., ε″_*kk*_ or ε″_*cf*_. Recall that a single Debye relaxation is narrowed down upon inversion to an extremely sharp delta distribution. The ensuing distribution spectra will therefore show the highest resolution possible. We will discuss a simple way to accomplish the complex Stieltjes inversion of the ε′ and ε″ data by making use of rational polynomials in fractional powers. A joint data-analysis by fitting ε′ and ε″ “apart-together” leads to an improvement in resolution power as well. Such a paired simultaneous modeling, dubbed *all-in-one* modeling, wherein the relaxation parameters are kept the same in the ε′ and ε″ fit functions, but whereby the conduction is contained only in the ε″ fit function, requires a two-way switch in the co-fit procedure which links the proper data to the proper fit formulae and thus assures that the two non-linear least squares minimizations are always done in parallel. Although we will focus on the data handling of the complex permittivity, the fast conversion methods developed can also be applied directly to other complex electrokinetic quantities like the dielectric modulus and the impedance or to the magnitude and phase.

Apart from the ohmic conduction, another disturbance is prominently active in the dielectric spectroscopy of aqueous colloidal systems. The point being that the ions of the electrolyte tend to accumulate near the electrodes of the measuring cell. This causes electrode polarization (EP), which may enhance ε′ and ε″ strongly. This phenomenon too may therefore overshadow the true l.f. relaxation of the colloidal particles. We will briefly describe in Section Improving the Resolution by All-In-1 Modeling of the Real and Imaginary Data, how the effect of this nuisance can be eliminated as well as. Several methods to accomplish this are discussed recently in more detail in an upcoming article of this journal (Chassagne et al., submitted) and in van Turnhout et al. (2016).

**Figure 3:**
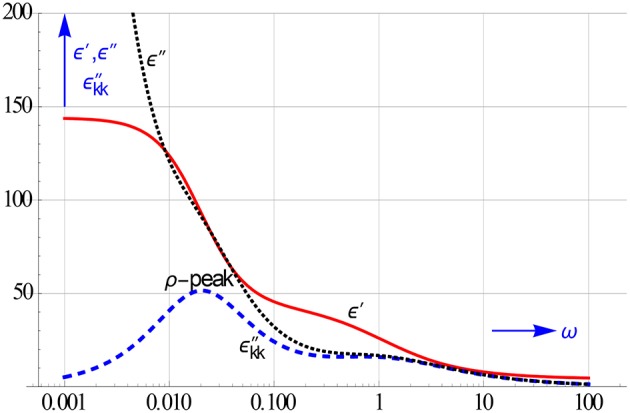
**The l.f. ρ or space charge relaxation, which is due to the rather slow up and down motion of the ion clouds near the electrodes, remains hidden in the ε″ curve**. It too can be disentangled from the conduction contribution by calculating ε″_*kk*_. The conduction, which stems from the gross ion motion, appears alongside the EP, if the electrodes are not fully blocking.

Figure 3 illustrates the impact of the EP. The local ion motions in tune with the a.c. voltage create a special relaxation peak, which we have called ρ or space charge peak. Clearly, the calculated $\varepsilon_{kk}^{\prime \prime }$ spectrum will also reveal the presence of this specific space charge relaxation peak.

**Figure 4:**
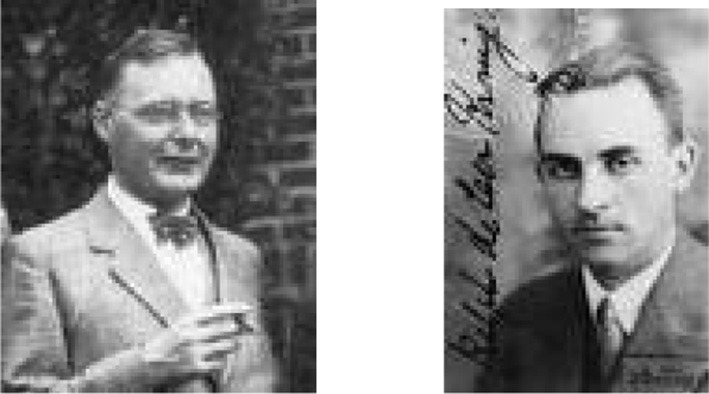
**Kramers (left), in particular Kronig (right) was relatively young when he proposed one of the KK integrals**. At that time he added de Laer to his last name. His name should not be spelled Krönig.

## KK relations-the basics of the interconversion of real and imaginary data

The KK dispersion relations were proposed by Kramers and Kronig about a century ago. A candid review of their history has been given by Bohren (2010). The relations were given as integrals that relate the real and imaginary part of all dispersion phenomena. Kramers proposed both integrals for the first time in Copenhagen (Kramers, 1927). Kronig proposed one of them a year later (Kronig, 1926). Both did not refer in their accounts to the Hilbert transform and it inverse, which are basically the same.

Electrical engineers speak of “real part sufficiency,” which implies that the imaginary part can be calculated from the real part of the response of an electric network. They present the integrals in their textbooks without reference to KK (see e.g., Turtle, 1958). It took some time before the KK relations were actually used in the research of dielectric phenomena. Early pioneers were for instance: Bayard (1935), Gorter and Kronig (1936), Gross (1943), Brachman and Macdonald (1954).

The KK relations can be derived by making use of Cauchy's integral theorem (Kremer and Schönhals, 2002). They have the following form for dielectric dispersions or relaxations

(2.1) $$\begin{array}{lll} \varepsilon^{\prime }\left(\omega_{o}\right)-\varepsilon_{\infty } & = & \frac{2}{\pi }\int_{0}^{\infty }\frac{\omega \varepsilon^{\prime \prime }\left(\omega \right)}{\omega^{2}-\omega_{o}^{2}}d\omega \\ \varepsilon^{\prime \prime }\left(\omega_{o}\right) & = & -\frac{2\omega_{o}}{\pi }\int_{0}^{\infty }\frac{\varepsilon^{\prime }\left(\omega \right)-\varepsilon_{\infty }}{\omega^{2}-\omega_{o}^{2}}d\omega , \end{array}$$

where ε′ and ε″ are the real and imaginary part of the complex permittivity ε^*^(ω) = ε′(ω) − iε″(ω). Similar integrals hold for the other quantities used to describe dielectric relaxations such as the complex dielectric modulus, m^*^(ω) = 1∕ε^*^(ω), the magnitude, |ε^*^(ω)|, and phase θ(ω) = atn[ε″(ω)/ε′(ω)]. We shall restrict ourselves mainly to the interconversion of ε′ to ε″ and vice versa. In this process we will derive so-called conversion frames that can equally well be used to convert m′ into m″, etc.

Considering the integrals given, it is not surprising that in practice the KK relations are still not broadly used. This is due to the fact that their kernels become singular for ω → ω_*o*_. However, this singularity can be removed by rewriting them to

(2.2) $$\begin{array}{lll} \varepsilon^{\prime }\left(\omega_{o}\right)-\varepsilon_{\infty } & = & \frac{2}{\pi }\int_{0}^{\infty }\frac{\omega \varepsilon^{\prime \prime }\left(\omega \right)-\omega_{o}\varepsilon^{\prime \prime }\left(\omega_{o}\right)}{\omega^{2}-\omega_{o}^{2}}d\omega \\ \varepsilon^{\prime \prime }\left(\omega_{o}\right) & = & -\frac{2\omega_{o}}{\pi }\int_{0}^{\infty }\frac{\varepsilon^{\prime }\left(\omega \right)-\varepsilon^{\prime }\left(\omega_{o}\right)}{\omega^{2}-\omega_{o}^{2}}d\omega . \end{array}$$

**Figure 5:**
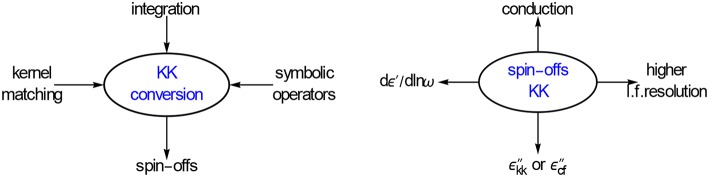
**On the left, numerical integration is not the favorite tool for KK conversion**. It can be better realized with kernel matching and with symbolic differential operators. The conversion produces useful spin offs, they are depicted on the right. The higher resolution results from the elimination of the conduction from the ε″-data.

Nonetheless the conversion of ε′ into ε″-values with an integral like Equation (2.2) is a tedious job. The more so because we want to know the converted values across the whole frequency scan. We have therefore discarded numerical integration and have followed two different approaches, see Figure 5.

Before outlining this in Sections Easy to Use Conversion Frames Obtained by Matching Debye Kernels and Computing Conversion Frames with Symbolic Differential Operators, we should point out an important salient property of the KK relations, which is often overlooked. By the very fact that ε″ can be calculated from ε′, we miss out any possible contribution by ohmic conduction. In other words by invoking the KK relations we get a special set of ε″-data that are conduction free. We should therefore label the converted data with cf or kk and denote them as $\varepsilon_{kk}^{\prime \prime }$ or $\varepsilon_{cf}^{\prime \prime }$. Since the ohmic conduction causes a loss component of $\varepsilon_{c}^{\prime \prime }\left(\omega \right)=\gamma ∕\left(\varepsilon_{o}\omega \right)$ we have

(2.3) $$\begin{array}{l} \varepsilon_{kk}^{\prime \prime }\left(\omega \right)=\varepsilon^{\prime \prime }\left(\omega \right)-\varepsilon_{c}^{\prime \prime }\left(\omega \right)=\varepsilon^{\prime \prime }\left(\omega \right)-\gamma ∕\left(\varepsilon_{o}\omega \right), \end{array}$$

where ε″(ω) represents the measured ε″-data, $\varepsilon_{c}^{\prime \prime }\left(\omega \right)$ the conductive part in ε″, γ the ohmic conductivity and ε_*o*_ the permittivity of vacuum.

The KK relations can be of help in the removal of the dissipative ohmic loss only from ε″. They cannot be recruited for its removal from the other dielectric quantities mentioned. Let us illustrate this for the magnitude-phase relation. Writing ε^*^(ω) in its polar Euler form

(2.4) $$\begin{array}{l} \varepsilon^{*}\left(\omega \right)\;=\;|\varepsilon^{*}\left(\omega \right)|e^{i\theta \left(\omega \right)} \end{array}$$

and taking the natural logarithm we get:

(2.5) $$\begin{array}{l} \text{ln}\left[\varepsilon^{*}\left(\omega \right)\right]=\ln\left[\varepsilon_{a}\left(\omega \right)\right]+\text{i}\theta \left(\omega \right), \end{array}$$

with ε_*a*_(ω) equaling the absolute value |ε^*^(ω)| and θ(ω) = atn[ε″(ω)/ ε′(ω)].

The real and imaginary parts lnε_*a*_ and θ obey KK relations similar to those of Equation (2.1). This means that it is possible to compute θ from lnε_*a*_ values, but this does not imply that in doing so the $\varepsilon_{c}^{\prime \prime }$ contribution to θ is nullified. The reason being that the real part lnε_*a*_ also contains a contribution from $\varepsilon_{c}^{\prime \prime }$. Recall that $\varepsilon_{a}\left(\omega \right)=\sqrt{\varepsilon^{\prime }{\left(\omega \right)}^{2}+\varepsilon^{\prime \prime }{\left(\omega \right)}^{2}}$, so ε′ and ε″ are now mixed up in the real part.

Zahner (Germany) has implemented in their software this “logarithmic” version of the KK relations (Schiller et al., 2001; Lasia, 2014). It embodies an algorithm to calculate lnε_*a*_ from measured θ values. We will return to this in Sect. 4. The logarithmic version of the KK relations is often used by electrical engineers (Turtle, 1958).

**Figure 6:**
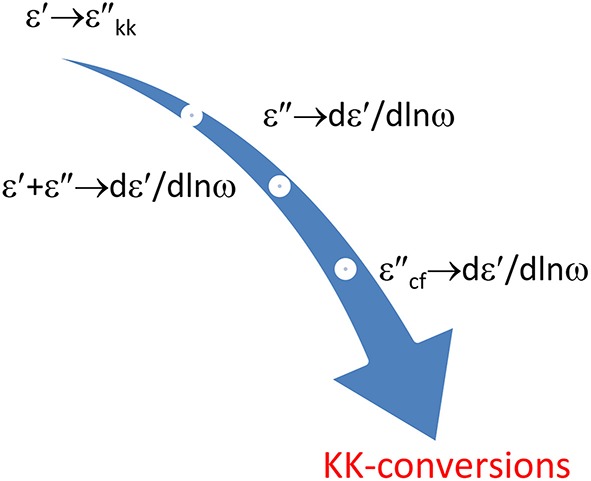
**Illustration of the different conversions covered**. The resulting ε″_*kk*_ and *dε*′∕*dlnω* will improve the l.f. spectral resolution, both with respect to ohmic conduction and to EP.

Figure 6 shows the various conversions we will discuss below. For each we will give the coefficients of the appropriate conversion frames. The main focus will be on getting one liners for the conversion of ε′ to ε″_*kk*_ and ε″ to *dε*′∕*dlnω*. Although, as just said, we will also shortly touch upon the conversion of θ to lnε_*a*_ or for that matter of ε″ to ε′.

## Easy to use conversion frames obtained by matching Debye kernels

The direct use of the unwieldy KK integrals can be avoided by making use of the integral equations based on the description of relaxation phenomena with a distribution in relaxation times.

The most elementary dielectric dispersion phenomenon is the Debye relaxation, which Debye derived for dipole relaxations in gases and liquids. We can view the intricate relaxation of colloidal systems as being buildup from a sum of Debye processes

(3.1) $$\begin{array}{l} \varepsilon^{*}\left(\omega \right)\;=\;\varepsilon_{\infty }\;+\;\sum_{\;k=1}^{\;n}\frac{\Delta \varepsilon_{k}}{1\;+\;i\omega \tau_{k}}, \end{array}$$

where ε_∞_ is ε′(∞), τ_*k*_ the relaxation time of process k. The relaxation strength Δε_*k*_ can be expressed as Δε_*k*_ = ε_*sk*_ − ε_∞_ with $\overset{n}{\underset{k=1}{\sum }}\varepsilon_{sk}=\varepsilon^{\prime }\left(0\right)$. Often the individual Debye processes may come very close, so we better go a step further and think of the total dielectric response arising from a continuous distribution of Debye processes f (τ)

(3.2) $$\begin{array}{l} \varepsilon^{*}\left(\omega \right)=\varepsilon_{\infty }+\Delta \varepsilon \int_{0}^{\infty }\frac{f\left(\tau \right)d\tau }{1+i\omega \tau }, \end{array}$$

in which we can split the complex Debye kernel into a real and imaginary part

(3.3) $$\begin{array}{l} \varepsilon^{\prime }\left(\omega \right)=\varepsilon_{\infty }+\Delta \varepsilon \int_{0}^{\infty }\frac{f\left(\tau \right)d\tau }{1+\omega^{2}\tau^{2}}\;\varepsilon^{\prime \prime }\left(\omega \right)=\Delta \varepsilon \int_{0}^{\infty }\frac{f\left(\tau \right)\omega \tau d\tau }{1+\omega^{2}\tau^{2}}. \end{array}$$

We can prove that these integrals obey the KK relations by inserting them into Equation (2.1).

We now have 2 additional relations between ε′ and ε″ that are linked up by the distribution function. Expressions like Equation (3.3) are common in the theory of viscoelastic phenomena. It were Ninomiya and Ferry (1959) who were the first to suggest a powerful trick to manipulate the various viscoelastic interrelations. By the way they did not deal with the interconversion of Equation (3.3) as such, but addressed e.g., the conversion of time to frequency responses and vice versa.

**Figure 7:**
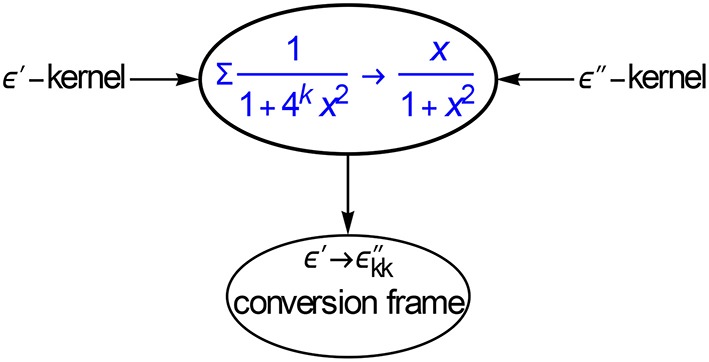
**Kernel matching is a powerful method for computing the coefficients of conversion frames, in this case for ε′ → ε″_*kk*_**. With the frames we can obtain ε″_*kk*_ with one liners, cf. Equations (3.7) and (3.8).

The clue, as sketched in Figure 7, is to approximate the respective Debye kernels. We have

(3.4) $$\begin{array}{l} \kappa^{\prime }\left(x\right)=\frac{1}{1+x^{2}}\;\kappa^{\prime \prime }\left(x\right)=\frac{x}{1+x^{2}}, \end{array}$$

where x = ωτ. Hence for the conversion of ε′ to ε″ we should approximate ε″ with a sum of κ′(x). A good option is

(3.5) $$\begin{array}{l} \kappa^{\prime \prime }\left(x\right)≃\sum_{k=-n}^{n}a_{k}\left[\kappa^{\prime }\left(2^{k}x\right)-1∕2\right]. \end{array}$$

The subtraction of ½ makes the κ′ kernel antisymmetric around *x* = 1 and assures that the a_*k*_'s become symmetric. By prescribing the logarithmic spacing of the κ′ terms at *h* = 2, we have reduced the least squares approximation to a linear one, which is easy to do. We found:

(3.6) $$\begin{array}{l} a_{k}=\{0.16192,-0.17918,0.30015,0.430378,0,-0.40378,\\ -0.30015,0.17918,-0.16192\} \end{array}$$

These coefficients differ somewhat from the ones, we have given earlier (Steeman and van Turnhout, 1997; Wübbenhorst and van Turnhout, 2002; van Turnhout, 2004). We call this set of coefficients a *conversion* frame. With such a frame or panel we can convert a set of log-spaced ε′ data easily to a $\varepsilon_{kk}^{\prime \prime }$value. In fact

(3.7) $$\begin{array}{l} \varepsilon_{kk}^{\prime \prime }\left(\omega \right)≃\sum_{k=-4}^{4}a_{k}\varepsilon^{\prime }\left(2^{k}\omega \right)\text{ or}\\ \varepsilon_{kk}^{\prime \prime }\left(\omega \right)≃\sum_{k=1}^{4}a_{4k}\left[\varepsilon^{\prime }\left(2^{-k}\omega \right)-\varepsilon^{\prime }\left(2^{k}\omega \right)\right], \end{array}$$

with a_4*k*_ = {0.40378, 0.300158, −0.17918, 0.16192}. The data acquisition of modern test equipment (e.g., from Novocontrol, HP-Agilent, now Keysight Technologies, etc) allow us to measure at log-spaced intervals, whereby the choice of a spacing *h* = 2 poses no problem.

Sampling the list of measured ε′ by sliding the frame from the beginning to the end step by step along all data creates a new set of ε″ data that are conduction free. We notice that for each ε″ value we need 4 ε′ data upfront and 4 at the rear. This means that we will lose 4 ε″ at the start and 4 at the end.

For that reason we have also calculated asymmetric conversion frames by starting the kernel-approximation of Equation (3.5) not from *k* = −4, but from −3, −2, −1. Using these start up frames we only miss the very first $\varepsilon_{kk}^{\prime \prime }$ value. By starting the sum in Equation (3.5) from *k* = −7, −6, −5, we get special end frames by which we lose out only one $\varepsilon_{kk}^{\prime \prime }$-value at the end of the frequency scan.

Most dielectric measurements cover a very wide ω range. Then the loss of a few ε″_*kk*_ data at the start and the end of the range is hardly serious. In that case we can restrict the conversion to that with the central frame and simply use Equation (3.7).

Table 1 lists the coefficients of the ε′ to ε″ frames. We have only included the central frame and those for the start, because those at the end are in fact the same as those at the start albeit in reversed order and with a minus-sign.

**Table 1:** **Coefficients of conversion frames for ε′ to $\varepsilon_{kk}^{\prime \prime }$**.

1.46798	−1.70423	1.22797	−1.42088	0.80091	−0.49497	0.18569	−0.05801	−0.00446
0.64739	−0.57793	1.26737	−1.60656	0.65318	−0.44159	0.10596	−0.02013	−0.02769
0.35083	−0.49787	1.15739	−0.45203	−0.33793	−0.0601	−0.17561	0.09753	−0.08221
0.16192	−0.17918	0.30015	0.40378	0	−0.40378	−0.30015	0.17918	−0.16192

Implemented in Mathematica, the frames with the coefficients b_1*k*_ to b_4*k*_ from top to bottom can be used as follows:

(3.8) $$\begin{array}{l} \text{Join}\left[\begin{array}{c} \text{Table}\left[\left\{\omega_{i},\overset{9}{\underset{k=1}{\sum }}{\text{b}}_{i-1,k}\varepsilon_{k}^{\prime }\right\},\left\{i,2,4\right\}\right],\\ \text{Table}\left[\left\{\omega_{i},\overset{4}{\underset{k=-4}{\sum }}{\text{b}}_{4,k+5}\varepsilon_{i+k}^{\prime }\right\},\left\{i,5,n-4\right\}\right],\\ \text{Table}\left[\left\{\omega_{i+n-5},-\overset{9}{\underset{k=1}{\sum }}{\text{b}}_{5-i,10-k}\varepsilon_{k+n-9}^{\prime }\right\},\left\{i,2,4\right\}\right] \end{array}\right]. \end{array}$$

At the start from ω_1_ we stick to the first 9 ε′ values to get ε″(2ω_1_), ε″(4ω_1_), ε″(16ω_1_). Next we can march on and drop the first ε′(ω_1_) and add a new ε′-value at the end of the central frame to get ε″(32ω_1_), ε″(64ω_1_), etc. This can be continued till we reach the end of the total of n ε′-data at ω_*n*_. We then stick to the last 9 ε′ data to get ε″[ω_*n*_/16], ε″[ω_*n*_/8], ε″[ω_*n*_/2]. The present frames give a more accurate conversion than the ones give a more accurate conversion than the ones given earlier (Steeman and van Turnhout, 1997).

**Figure 8:**
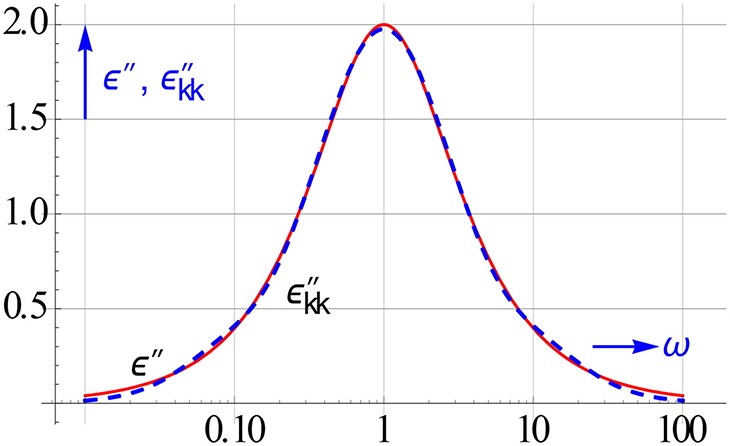
**KK conversion tested for a Debye relaxation with Equation (3.7)**. The dashed line pertains to ε″_*kk*_ calculated.

Figure 8 shows the accuracy achieved with the central panel for the ε″ data of a Debye relaxation (the sharpest relaxation possible). The recalculated values deviate a bit at the wings. This deviation becomes much less if the begin and end panels are used as well, as suggested in Equation (3.8).

Brather has also discussed KK conversion based on kern matching (Brather, 1979). He proposed the use of a series of ε′ terms. These large sums can be applied less easily used than our conversion frames, which allow conversion on line. He has also not discussed the special sums needed to begin and finish the conversion.

We should realize that the ohmic conduction will not contribute at all at the high end of the frequency range, because its loss $\varepsilon_{c}^{\prime \prime }$ drops off with 1/ω. At the end we may therefore also fill in the missing $\varepsilon_{kk}^{\prime \prime }$ with the measured ε″ values and thus skip the use of the special end frames.

In Steeman and van Turnhout (1997) we have also carried out the KK conversion of ε′ to ε″ by solving iteratively a triangular set of ε″ terms. It will be clear that the one-liners of Equations (3.7) and (3.8) are much easier to implement in the data analysis.

**Figure 9:**
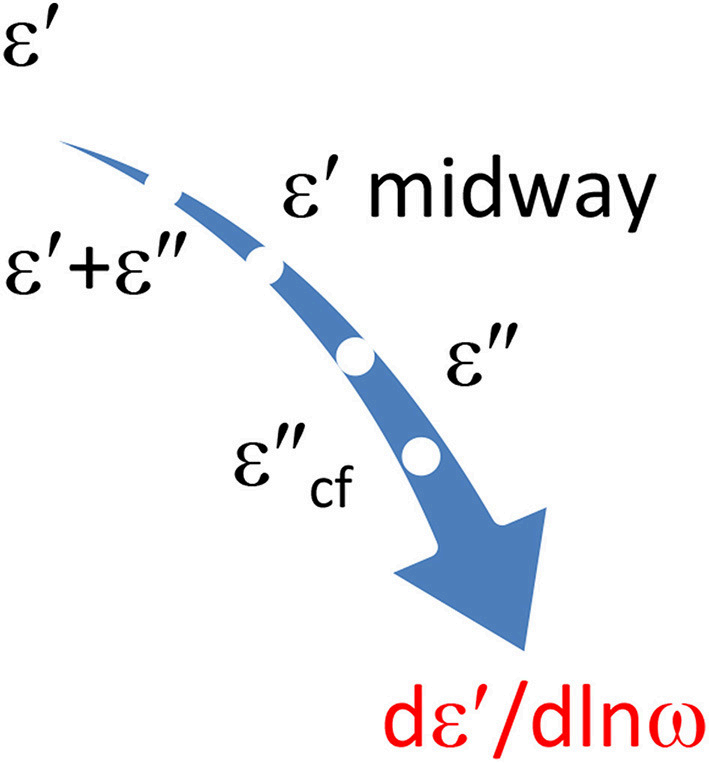
**A few options to obtain *dε*′∕*dlnω* via KK conversion**. Any contribution of possible conduction loss should of course be avoided if we recruit ε″ data. This can be effected by implying ε″_*cf*_ values. This special option is obtained by invoking a constraint. ε′ midway refers to sampling of the ε′ data at half the interval, cf. Equation (7.1). It produces a higher accuracy than the central discretization of ε′ with Equation (3.11).

Clearly, the calculation of conversion frames by kernel matching can easily be extended to other conversions. Such as the conversion of a few ε″ values to *dε*′∕*dlnω*. a mixed conversion of ε″ and ε′ to *dε*′∕*dlnω* is possible, possible, see Figure 9.

The logarithmic derivative of ε′ is a compelling quantity, because it has a higher resolution of nearby peaks than ε″. However, we should be careful not to infect the resulting *dε*′∕*dlnω* by ohmic conduction from ε″. This can easily be prevented by imposing a constraint on the linear l.s.q. minimization of the *dε*′∕*dlnω* kernel. We therefore matched

(3.9) $$\begin{array}{lll} \frac{d\kappa^{\prime }\left(x\right)}{d\ln\;x} & = & \frac{2x^{2}}{{\left(1+x^{2}\right)}^{2}}≃\sum_{k=-1}^{1}b_{k}\left[\kappa^{\prime }\left(2^{k}x\right)-1∕2\right]+\\ \overset{1}{\underset{k=-1}{\sum }}c_{k}\kappa^{\prime \prime }\left(2^{k}x\right), \end{array}$$

with as constraint $\overset{1}{\underset{k=-1}{\sum }}c_{k}\left(2^{-k}∕x+2^{k}x\right)=0$. This leads to the following mixed conversion

(3.10) $$\begin{array}{lll} d\varepsilon^{\prime }∕d\ln\;\omega & ≃ & 0.378359\left[\varepsilon^{\prime }\left(\omega ∕2\right)-\varepsilon^{\prime }\left(2\omega \right)\right]+0.595523\\ \left[-\varepsilon^{\prime \prime }\left(\omega ∕2\right)+2.5\varepsilon^{\prime \prime }\left(\omega \right)-\varepsilon^{\prime \prime }\left(2\omega \right)\right]. \end{array}$$

This mixed conversion illustrates the versatility of kernel matching. Yet it is still compatible with the KK relations. Apparently, we only need 2 ε′ and 3 ε″ values to get an accurate estimate of *dε*′∕*dlnω*.

Admittedly, we can use as alternative to Equation (3.9) the 5 term central logarithmic difference of ε′. This gives:

(3.11) $$\begin{array}{l} d\varepsilon^{\prime }∕d\ln\;\omega ≃\sum_{\;k=-2}^{\;2}d_{k}\varepsilon^{\prime }\left(h^{k}\omega \right), \end{array}$$

with d_*k*_ = {−1, 8, 0, −8, 1}/(12lnh). This numerical derivative, which derives from a 3rd degree logarithmic polynomial, is for *h* = 2 less accurate than (3.10) for sharp Debye like peaks.

**Figure 10:**
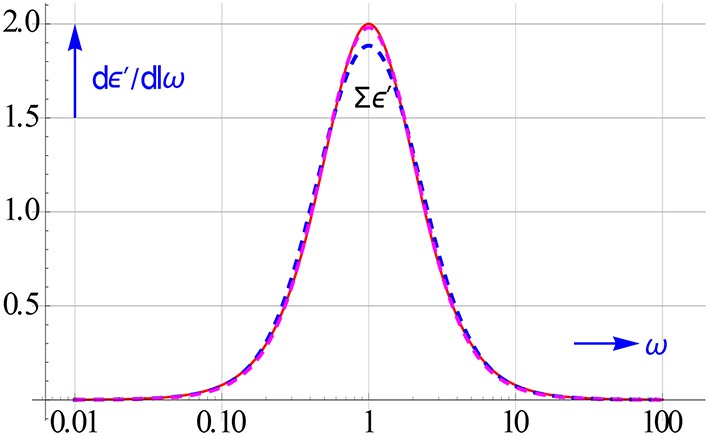
**Accuracy of 3 options for computing *dε*′∕*dlnω* for a Debye relaxation**. The commonly used central frame of Equation (3.11) is evidently not the best near the maximum for *h* = 2. The offset at the maximum disappears if we reduce h to 10^0.2^.

The accuracy obtained with 3 options for calculating *dε*′∕*dlnω* is demonstrated in Figure 10. The central difference frame performs less than the mixed frame of Equation (3.9) and also less than the “halfway” frame of Equation (7.1). An interval of *h* = 2 roughly equals 10^0.3^, taking *h* = 10^0.2^ improves the accuracy of the central difference markedly. It then scores virtually as good as the other two.

## Computing conversion frames with symbolic differential operators

There is a saying that one can do “integration by differentiation,” this also applies to the KK relations. The KK integrals are in fact logarithmic convolution integrals. This can be shown be rewriting Equation (2.1) to

(4.1) $$\begin{array}{lll} \varepsilon^{\prime \prime }\left(\omega_{o}\right) & = & -\frac{2\omega_{o}}{\pi }\int_{0}^{\infty }\frac{\varepsilon^{\prime }\left(\omega \right)-\varepsilon_{\infty }}{\omega^{2}-\omega_{o}^{2}}d\omega \\ =-\frac{2}{\pi }\int_{0}^{\infty }\frac{\varepsilon^{\prime }\left(u\omega_{o}\right)-\varepsilon_{\infty }}{u^{2}-1}du, \end{array}$$

in which ε′(*uω*_*o*_) now depends on the product of u and ω_*o*_. Integrals with such a function are called convolution integrals. They can be evaluated in a special way (Hirschman and Widder, 1955).

For pursuing this, we introduce the logarithmic derivative D_*l*_. This allows us to move the x-position of a function f to hx using the symbolic exponential operation

(4.2) $$\begin{array}{l} e^{D_{l}\ln\;h}f\left(x\right)=f\left(hx\right). \end{array}$$

This is the logarithmic variant of a better known operation with the normal derivative D, which produces a linear shift

(4.3) $$\begin{array}{l} e^{hD}f\left(x\right)=f\left(x+h\right). \end{array}$$

Equation (4.2) can be proven be developing both sides in a Taylor expansion around *h* = 1.

The exponential logarithmic derivative operation of Equation (4.2) can be replaced by a logarithmic shift E_*l*_

(4.4) $$\begin{array}{l} E_{l}f\left(x\right)=f\left(hx\right), \end{array}$$

which produces the equality

(4.5) $$\begin{array}{l} E_{l}=e^{D_{l}\ln\;h}\text{ or }D_{l}=\ln\;\left(E_{l}\right)∕\ln\;h. \end{array}$$

**Figure 11:**
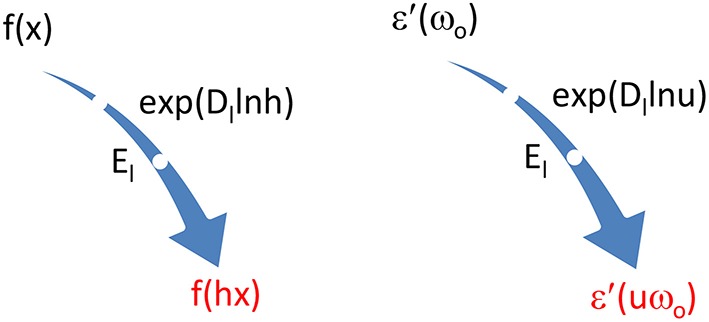
**On the left, two symbolic tools to transform f(x) to f(hx)**. On the right, trick to link ε′(uω_*o*_) symbolically to ε′(ω_*o*_) and thus push the latter out of the convolution integral of KK in Equation (4.8).

The action of both logarithmic operators for transforming f(x) into f(hx) is portrayed in Figure 11 on the left.

It may be illuminating to use the symbolic equality of Equation (4.5) to find the panel of coefficients for the 1st derivative of ε′: D_*l*_ε′ = *dε*′∕*dlnω*. Expanding lnE_*l*_ in a series of up to the 4th order around E_*l*_ = 1 results in

(4.6) $$\begin{array}{l} D_{l}=E_{l}^{2}\frac{\ln\;E_{l}}{\ln\;h}≃\frac{E_{l}^{2}}{\ln\;h}\sum_{k=-2}^{2}d_{k}E_{l}^{k}, \end{array}$$

with the coefficients d_*k*_ equaling those given in Equation (3.11). Note that we pre-multiplied with $E_{l}^{2}$ in order to get the coefficients of the central difference. Series expansions like that of Equation (4.6) are easy to do in symbolic programs like Maple and Mathematica.

With the help of e Equation (4.2), as sketched on the right in Figure 11, we may replace ε′(*uω*_*o*_) by

(4.7) $$\begin{array}{l} \varepsilon^{\prime }\left(u\omega_{o}\right)=e^{D_{l}\ln\;u}\varepsilon^{\prime }\left(\omega_{o}\right), \end{array}$$

inserting this in Equation (4.1) gives

(4.8) $$\begin{array}{l} \varepsilon^{\prime \prime }\left(\omega_{o}\right)=-\frac{2}{\pi }\int_{0}^{\infty }\frac{e^{D_{l}\ln\;u}}{u^{2}-1}du\left[\varepsilon^{\prime }\left(\omega_{o}\right)-\varepsilon_{\infty }\right], \end{array}$$

in other words we can push ε′(ω_*o*_)−ε_∞_ out of the KK-integral. The resulting integral with u as variable is known analytically. We thus get

(4.9) $$\begin{array}{l} \varepsilon^{\prime \prime }\left(\omega \right)=\tan\left(\pi D_{l}∕2\right)\left[\varepsilon^{\prime }\left(\omega \right)\right]. \end{array}$$

We speak of a *symbolic* differential operator. In a similar way we find as symbolic differential operator for the ε″ to ε′ conversion

(4.10) $$\begin{array}{l} \varepsilon^{\prime }\left(\omega \right)=-\cot\left(\pi D_{l}\;∕\;2\right)\left[\varepsilon^{\prime \prime }\left(\omega \right)\right]. \end{array}$$

We have thus indeed succeeded in replacing the KK integrals by KK differential operators.

Admittedly, the KK relations can be applied for the analysis of dispersions in many areas. One of these areas are ultrasonic relaxation studies, where their differential forms did not remain unnoticed (Waters et al., 2003).

**Figure 12:**
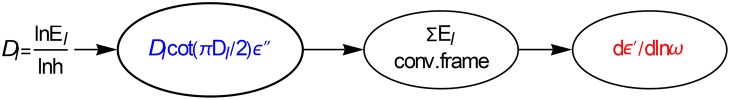
**Inserting D_*l*_ = lnE_*l*_/ln2 in a symbolic operator provides a versatile tool to get an expansion in E_*l*_, which in turn provides an easy-to-use conversion frame, in this case for ε″ to *dε*′∕*dlnω***.

The use of these unusual symbolic operators looks at first sight not easy. The job was facilitated a lot by inserting for D_*l*_ = lnE_*l*_/lnh in the tan and cot operator. Let us illustrate this for Equation (4.10), see also Figure 12. If we take the logarithmic derivative of both sides, we get

(4.11) $$\begin{array}{l} D_{l}\varepsilon^{\prime }\left(\omega \right)=d\varepsilon^{\prime }∕d\ln\;\omega =D_{l}\cot\left(\pi D_{l}∕2\right)\left[\varepsilon^{\prime \prime }\left(\omega \right)\right]. \end{array}$$

In order to arrive at a conversion frame for 5 logarithmically spaced ε″-data with *h* = 2, we expand Equation (4.11) as follows around E_*l*_ = 1

(4.12) $$\begin{array}{l} E_{l}^{2}\ln\;E_{l}∕\ln\;2\cot\left[\pi \ln\;E_{l}∕\left(2\ln\;2\right)\right]≃E_{l}^{2}\sum_{k=-2}^{2}e_{k}E_{l}^{k}, \end{array}$$

which yields for which yields for *dε*′∕*dlnω*,

(4.13) $$\begin{array}{l} d\varepsilon^{\prime }∕d\ln\;\omega ≃\sum_{\;k=-2}^{\;2}e_{k}\varepsilon^{\prime \prime }\left(2^{k}\omega \right) \end{array}$$

We did a similar expansion for only 3 ε″ terms. The *dε*′∕*dlnω* calculated with neither the 3 nor the 5 terms was satisfactorily accurate for a Debye relaxation (one produced an undershoot, the other an overshoot). We therefore took the average of the coefficients. This average results in

$$\begin{array}{l} {\text{e}}_{k}=\left\{-0.14821,-0.49697,1.9270,-0.49697,-0.14821\right\} \end{array}$$

with these coefficients the *dε*′∕*dlnω* calculated with Equation (4.13) turns out to be optimal.

We should stress that Equation (4.13) should *not* be used if the ε″-data contain ohmic conduction. But like kernel matching, the route via differential operators can be corrected for that. We will return to this in Section Simple Routes to a Higher Spectral Resolution: Logarithmic Derivatives or Differences of ε′ and ε″. We strongly recommend to rather use the frame from that section to convert measured ε″ data to *dε*′∕*dlnω*.

Being less ambitious, we could limit ourselves to using only just the very 1st term of the expansion of *D*_*l*_cot(π*D*_*l*_∕2)in D_*l*_ near 0. This equals 2/π, which gives as crude approximation

(4.14) $$\begin{array}{l} d\varepsilon^{\prime }∕d\ln\;\omega ≃2\varepsilon^{\prime \prime }\left(\omega \right)∕\pi . \end{array}$$

This approximation merely gives a reasonable estimate of *dε*′∕*dlnω* for broad relaxations. This modest zero-order estimate for *dε*′∕*dlnω* is furthermore not conduction free.

We mentioned in Section Introduction that Zahner (Germany) has incorporated a KK conversion in their software, viz. for calculating lnε_*a*_(ω) from an integral of the phase θ(ω) (Schiller et al., 2001; Lasia, 2014). This conversion follows directly from Equation (4.10) by expanding the cot operator in D_*l*_ up to the 1st term

(4.15) $$\begin{array}{l} \varepsilon^{\prime }\left(\omega \right)≃\left[2∕\left(\pi D_{l}\right)-\pi D_{l}∕6\right]\varepsilon^{\prime \prime }\left(\omega \right). \end{array}$$

Like the inverse of D, 1/D, the inverse operator 1/Dl stands for integration, in this case logarithmic integration. Realizing that lnε_*a*_ corresponds to ε′ and θ to ε″ we get

(4.16) $$\begin{array}{l} \ln\;\varepsilon_{a}\left(\omega_{o}\right)≃\frac{2}{\pi }\int_{\omega_{o}}^{\infty }\theta \left(\omega \right)d\ln\;\omega -\frac{\pi d\theta \left(\omega_{o}\right)}{6d\ln\;\omega }. \end{array}$$

The full integration of the θ values down to ω_*o*_ can be avoided if we calculate the logarithmic difference of lnε_*a*_. A close approximation for this difference can be derived by series matching not the cot-operator itself, but the cot operator minus the integration operator

(4.17) $$\begin{array}{l} \left(E_{l}^{-1}-E_{l}\right)\left[\cot\left(\pi D_{l}∕2\right)-2∕\left(\pi D_{l}\right)\right]. \end{array}$$

The pre-multiplication with 1/E_*l*_-E_*l*_ is required because

(4.18) $$\begin{array}{l} \Delta \varepsilon^{\prime }\left(\omega \right)=\varepsilon^{\prime }\left(\omega ∕2\right)-\varepsilon^{\prime }\left(2\omega \right)=\left(E_{l}^{-1}-E_{l}\right)\varepsilon^{\prime }\left(\omega \right). \end{array}$$

We have done the series matching in a special way by matching to a sum in E${}_{l}^{2}$ rather than E_*l*_. The reason being that this gives a more accurate approximation. This leads to

(4.19) $$\begin{array}{ll} \left(E_{l}^{-1}-E_{l}\right)\left\{\cot\left[\pi \ln\;E_{l}∕\left(2\ln\;h\right)\right]-2\ln\;h∕\left(\pi \ln\;E_{l}\right)\right\}\\ ≃ & \sum_{k=-1}^{1}a_{k}E_{l}^{2k}, \end{array}$$

which gives *a*_*k*_ = {−1, 2, −1}π/(12lnh) and results for Δlnε_*a*_ with *h* = 2 in

(4.20) $$\begin{array}{lll} \Delta \ln\;\varepsilon_{a}\left(\omega_{o}\right) & = & \ln\;\varepsilon_{a}\left(\omega_{o}∕2\right)-\ln\;\varepsilon_{a}\left(2\omega_{o}\right)≃\frac{2}{\pi }\overset{2\omega_{o}}{\underset{\omega_{o}∕2}{\int }}\theta \left(\omega \right)d\ln\;\omega \\ +\sum_{k=-1}^{1}a_{k}\theta \left(4^{k}\omega_{o}\right). \end{array}$$

By combining this with the logarithmic version of the Simpson rule we get

(4.21) $$\begin{array}{l} \Delta \ln\;\varepsilon_{a}\left(\omega \right)≃\frac{2\ln\;2}{3\pi }\left[\theta \left(\omega ∕2\right)+4\theta \left(\omega \right)+\theta \left(2\omega \right)\right]+\sum_{k=-1}^{1}a_{k}\theta \left(4^{k}\omega \right). \end{array}$$

Checking Equation (4.21) for a Debye relaxation, we found that we could improve the results by multiplying the a_*k*_ coefficients with 0.94. This produces for *h* = 2 the following conversion

(4.22) $$\begin{array}{l} \Delta \ln\;\varepsilon_{a}\left(\omega \right)≃\sum_{k=-2}^{2}a_{sk}\theta \left(2^{k}\omega \right), \end{array}$$

with *a*_*sk*_ = {−0.355035, 0.14709, 1.29843, 0.14709, −0.355035}. If we wish we can use Equation (4.22) recursively and obtain by starting from a given lnε_*a*_ value at high ω, values for lnε_*a*_ at successively lower ω's.

This new approximation for lnε_*a*_ can clearly also be used to convert ε″-data to Δε′(ω) ones

(4.23) $$\begin{array}{l} \Delta \varepsilon^{\prime }\left(\omega \right)=\varepsilon^{\prime }\left(\omega ∕2\right)-\varepsilon^{\prime }\left(2\omega \right)≃\sum_{k=-2}^{2}a_{sk}\varepsilon^{\prime \prime }\left(2^{k}\omega \right). \end{array}$$

An interesting spin off of this conversion of ε″ to Δε′(ω) is that it allows us to calculate the ε′ response of models of which only ε″ is analytically known. Two typical examples are the Fuoss-Kirkwood and Jonscher models (see e.g., Tschoegl, 1989; Kremer and Schönhals, 2002).

There is hardly any need to use Equation (4.23) for converting measured ε″ data to Δε′. However, if we do, then we should realize that casual ohmic conduction may contaminate the Δε′ values, because the conversion frame is not conduction free. We mentioned above that by contrast the presence of ohmic conduction does not affect the proper conversion of θ to Δlnε_*a*_.

By employing kernel matching with constrains we have found a conversion frame in which any conduction loss is canceled. This reads

(4.24) $$\begin{array}{l} \Delta \varepsilon^{\prime }\left(\omega \right)≃\sum_{k=-2}^{2}a_{fk}\varepsilon^{\prime \prime }\left(2^{k}\omega \right), \end{array}$$

with *a*_*fk*_ = {−0.485235, 0.465031, 0.899672, 0.465031, −0.485235}. Imposing the conduction free constraint causes the conversion to Δε′ to become a bit less accurate.

Surely, we could have found the frame for converting ε″ to Δε′values also directly from the cot operator itself, by simply matching

(4.25) $$\begin{array}{l} E_{l}\left(E_{l}^{-1}-E_{l}\right)\cot\left[\pi \ln\;E_{l}∕\left(2\ln\;2\right)\right]≃E_{l}\sum_{k=-1}^{1}a_{ck}E_{\;l}^{\;k}. \end{array}$$

This gives a 3 term frame, we combined it with a 5 term frame to get optimal results for a Debye relaxation. This yields a combined frame with the following coefficients

*a*_*ck*_ = {−0.421343, 0.321676, 1.08188, 0.321676, −0.421343}. It turned out that the frame based on *a*_*sk*_ performs somewhat better to find Δε′ from ε″ than *a*_*ck*_. Overall the panel of *a*_*sk*_ shows the highest accuracy for the KK-conversion of ε″ to Δε′.

Shtrauss has also discussed the use of conversion frames (Shtrauss, 2005, 2006). He derives the frames via the Mellin transform. This approach has some resemblance with the symbolic route. Shtrauss calculated his frames (which he calls functional filters) with l.s.q. If he would have used series expansion then he would have found the same coefficients as we have presented in our symbolic panels.

## Uncovering the l.f. dispersion by calculating the conduction free ε″ losses

The removal of the contribution by ohmic loss to the l.f. dispersion has become an easy task with the availability of a fast KK conversion of ε′ to ε″. We just have to include the conversion frames of Table 1 in the data handling. They require just a few lines of code as Equation (3.8) shows. In particular the central part of the $\varepsilon_{kk}^{\prime \prime }$ data is easily computed with Equation (3.7).

The ohmic conduction originates from the continuous flow of ions toward the electrodes. A flow that is driven by the applied a.c. field. In some colloidal systems percolation might happen at a certain critical concentration, when the conducting phase becomes co-continuous. This will show up in a sharp rise in the ohmic conduction. This can be monitored by calculating the conduction from the difference between the observed ε″ and the computed $\varepsilon_{kk}^{\prime \prime }$, by using Equation (2.3) in reverse

(5.1) $$\begin{array}{l} \gamma ∕\left(\varepsilon_{o}\omega \right)=\varepsilon^{\prime \prime }\left(\omega \right)-\varepsilon_{kk}^{\prime \prime }\left(\omega \right). \end{array}$$

**Figure 13:**
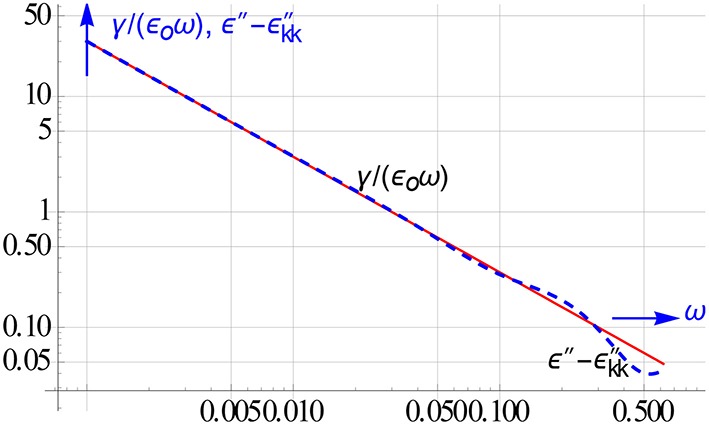
**One of the spin offs of the KK conversion, the retrieval of the conduction from the measured and the converted ε″ data**.

Figure 13 displays the result of the computed conduction for a Debye relaxation with conduction loss present. The conduction is recovered closely up to a quite high frequency.

## Conversion without KK by approximating ε′ and ε″ data with a complex rational function

In commercial software the KK conversion is avoided by assuming that the data can be modeled with a complex empirical function, ε′ and ε″ of which can each be specified explicitly. Usually the HN function is preferred for this purpose (Kremer and Schönhals, 2002). It reads

(6.1) $$\begin{array}{l} \varepsilon_{hn}^{*}\left(\omega \right)=\varepsilon_{\;\infty }+\frac{\Delta \varepsilon }{{\left[1+{\left(i\omega \tau \right)}^{\;a}\right]}^{\;b}}-\frac{\;i\gamma }{\varepsilon_{o}\omega }, \end{array}$$

in which the last term accounts for ohmic conduction. The HN function has two shape or peak broadening parameters a and b. It can thus model the real and imaginary part of the experimental data of a relaxation phenomenon often successfully. Usually, a and b are assumed to remain below 1, but the proper constraints are 0 < a < 1 and 0 < ab < 1. The latter implies that b may exceed 1.

Imposing a specific model function is unsatisfactory, because it does not derive from the underlying process. This pertains the more so, if the system under study embodies a variety of relaxation phenomena. This is in fact often the case for colloidal systems (Grosse, 2002; Chassagne and Bedeaux, 2008; Delgado et al., 2014).

**Figure 14:**
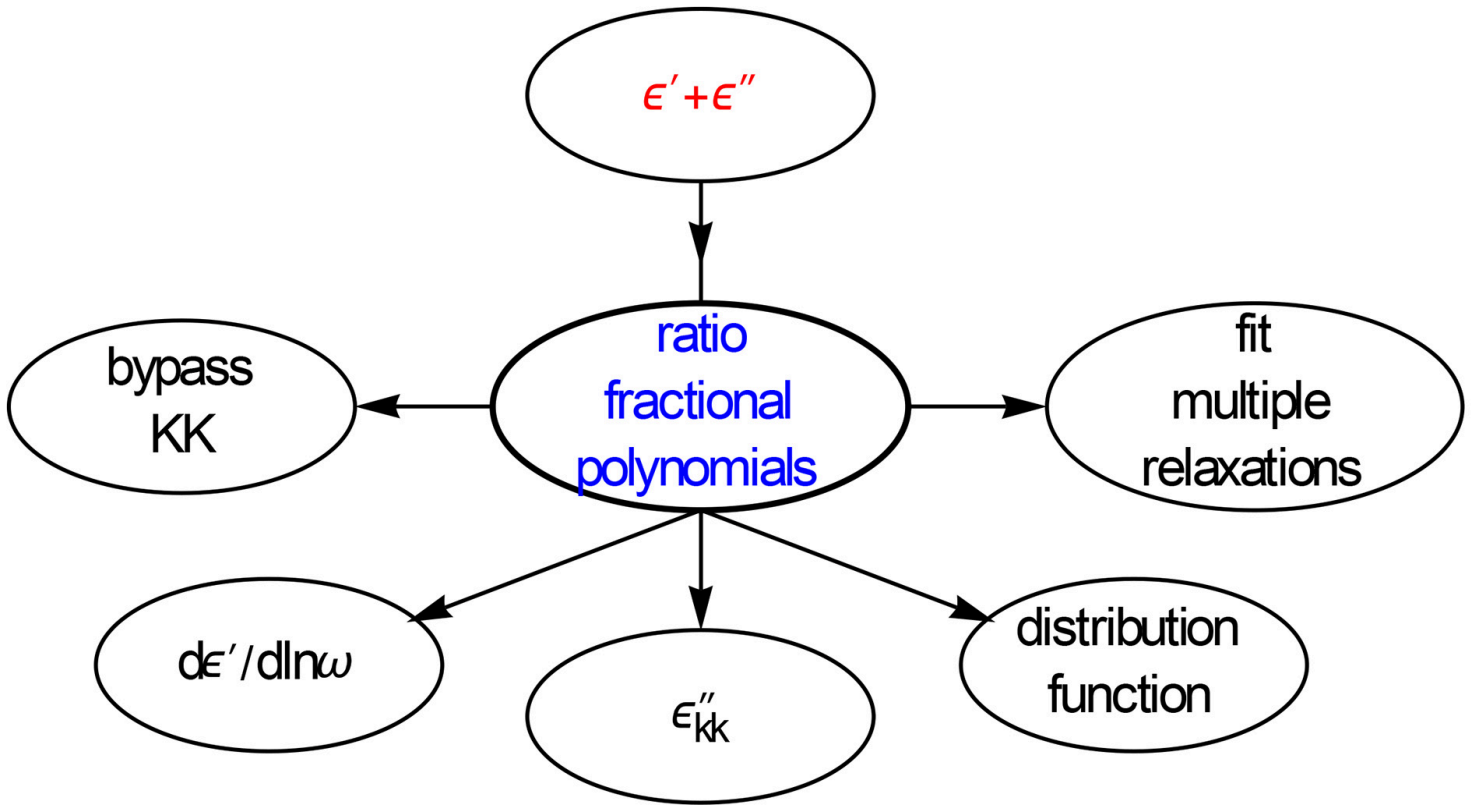
**A ratio of complex fractional sums provide a convenient means to model relaxation spectra**. Multiple peaks pose no problem and we get easy access to conduction free ε″ data and *dε*′∕*dlnω* without any involvement of the KK relations.

We have therefore followed a different model free approach and have approximated ε′ and ε″ with the real and imaginary part of a complex ratio in fractional power sums. We have also included a conduction term. The leads to the following expression for ε^*^

(6.2) $$\begin{array}{lll} \varepsilon^{*}\left(\omega \right) & = & \varepsilon_{\infty }+\varepsilon_{r}^{*}\left(\omega \right)-i\frac{\gamma }{\varepsilon_{o}\;\omega }=\varepsilon_{\infty }\\ + & \frac{\sum_{k=0}^{n}a_{k}{\left(i\omega \right)}^{ck}}{1+\sum_{\;k=1}^{\;n+1}b_{k}{\left(i\omega \right)}^{ck}}-i\frac{\gamma }{\varepsilon_{o}\;\omega }, \end{array}$$

where c is a fractional power and ε${}_{r}^{*}$ represents the dispersion or relaxation part. $-ℑ\left[\varepsilon_{r}^{*}\left(\omega \right)\right]$ now plays the role of ε″_*kk*_. An upper limit of *n* = 2 is often sufficient. The possibilities offered by approximating ε′ and ε″ data with a ratio of complex fractional polynomials in ω are indicated in Figure 14,^2^.

We could model ε′ and ε″ spectra with several relaxation peaks quite accurately with Equation (6.2). We usually started the l.s.q. minimization with *c* = 0.5 as initial value. The capability of Equation (6.2) to model multiple peaks in one go, is due to the fact that we do not need to include a relaxation time like we have to do for the common empirical functions like that of HN. They are meant to fit one peak only, whereby the relaxation time τ dictates the peak position on the scale (ω_*m*_ about 1/τ). We should take care to put in Equation (6.2) the degree in the degree in the denominator one higher than that of the numerator.

In order to recover both the relaxation part ε${}_{r}^{*}$ and the conduction term we have combined a two component input (the ε′ and ε″ data) with a two way fit [viz. to the real and imaginary part of Equation (6.2)] when the observed relaxation spectra show multiple peaks. We further advise to fit instead to the featureless ε′ or in addition to it the unstructured ε′, logarithmic difference Δε′(ω), defined by

(6.3) $$\begin{array}{l} \Delta \varepsilon^{\prime }\left(\omega \right)=\varepsilon^{\prime }\left(\omega ∕2\right)-\varepsilon^{\prime }\left(2\omega \right)=ℜ\left[\varepsilon_{r}^{*}\left(\omega ∕2\right)-\varepsilon_{r}^{*}\left(2\omega \right)\right]. \end{array}$$

Further details of this new joint multifunctional fit will be given in Section Improving the resolution by all-in-1 modeling of the real and imaginary data.

After this what we call all-in-1 modeling, ε″_*kk*_ simply follows from

(6.4) $${\varepsilon^{\prime \prime }}_{kk}(\omega )=-ℑ[\varepsilon_{r}^{*}(\omega )].$$

The ε″_*kk*_ obtained from the fractional power approximation covers directly the whole ω range, no special calculations are needed at the start and the end of the range.

Having an analytical expression for ε^*^, we can also use Equation (6.2) to calculate its logarithmic derivative. This can be given in closed form, the real part of which leads to

(6.5) $$\begin{array}{l} \frac{d\varepsilon^{\prime }}{d\ln\;\omega }=-ℜ\frac{c\overset{\text{m}+\text{n}}{\underset{j=0}{\sum }}{\left(i\omega \right)}^{cj}\overset{\min\left(j,m\right)}{\underset{k=\max\left(0,j-\text{n}\right)}{\sum }}\left(2k-j\right)a_{k}b_{j-k}}{\overset{2n}{\underset{j=0}{\sum }}{\left(i\omega \right)}^{cj}\overset{\min\left(j,\text{n}\right)}{\underset{k=\max\left(0,j-\text{n}\right)}{\sum }}b_{k}b_{j-k}}, \end{array}$$

where m is the upper limit in the numerator sum of ε^*^, and n that in the denominator sum. We may usually take n = m + 1, with m say *m* = 2. The use of the all-in-1 l.s.q. fit as the basis to obtain ε″_*kk*_ and *dε*′∕*dlnω*, has the advantage that these quantities become less prone to experimental errors.

**Figure 15:**
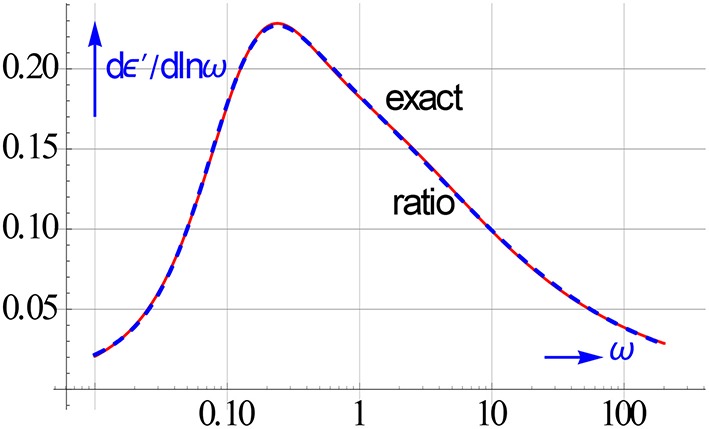
**After an all-in-1 rational fit *dε*′∕*dlnω* is directly analytically available from Equation (6.5)**. The accuracy of such a l.s.q. based *dε*′∕*dlnω* is clearly high. It coincides with the exact curve across a broad ω range.

The accuracy reached with Equation (6.5) and *m* = 2 and *n* = 3 for the merger of a double HN relaxation with ε_∞_ = 0.6, Δε_1_ = 0.75, a_1_ = 0.6, b_1_ = 0.7, τ_1_ = 1, Δε_2_ = 0.5, a_2_ = 0.9, b_2_ = 0.7, and τ_2_ = 7 is shown in Figure 15. We see a nice lining up with the exact *dε*′∕*dlnω* curve. This holds by the way also for the fits to the ε′ and ε″ data. It is interesting to note that the *dε*′∕*dlnω* curve with its better resolution indeed hints, albeit it vaguely, to the presence of two relaxations.

We successfully tried some other model free approximations for ε^*^. These too allowed us to go around the KK conversion; they will be discussed elsewhere. The fractional power approximation for ε^*^ of Equation (6.2) will be used in Section Improving the Resolution by All-In-1 Modeling of the Real and Imaginary Data to obtain a close analytical approximation to the distribution function of the underlying relaxation processes.

## Simple routes to a higher spectral resolution: logarithmic derivatives or differences of ε′ and ε″

The removal of the ohmic conduction from ε″ is essential for getting insight in the low frequency part of the dielectric spectrum. But also the rest of the spectrum contains a lot of information about the diversity of processes possible. They occur alongside each other, and they often overlap.

We will obtain the best fingerprint if the various phenomena are resolved as good as possible. The most common way is to consider the ε″ spectra. For sure this demonstrates with its peaks much more structure than the monotonous ε′-spectra. But better options are at hand. The derivative *dε*′∕*dlnω* e.g., has a better resolution than ε″. However, an accurate numerical calculation of *dε*′∕*dlnω* from *h* = 2 spaced ε′-data is less easy than it seems for sharp peaks. Since most colloidal systems are liquids most of their relaxations are rather sharp (i.e., Debye like).

In addition to the options to compute *dε*′∕*dlnω* presented in Sections Computing Conversion Frames with Symbolic Differential Operators and Uncovering the l.f. Dispersion by Calculating the Conduction Free ε″ Losses, we like to draw attention to the use the midway central difference. This can be obtained by expanding

(7.1) $$\begin{array}{l} D_{l}=E_{l}^{3∕2}\ln\;E_{l}∕\ln\;h=E_{l}^{3∕2}\sum_{k=-3∕2}^{3∕2}d_{hk}E_{l}^{k}, \end{array}$$

around E_*l*_ = 1, which gives d_*hk*_ = {−1, 27, −27, 1}/(24lnh). The higher accuracy of the midway logarithmic central difference for sharp peaks is due to the fact that it is based on ε′'s closer to the peak than the normal central difference Equation (4.6). That the halfway difference is more accurate has also been pointed out by Shtrauss (2006). The high accuracy of the half-spaced difference for *dε*′∕*dlnω* with *h* = 2 has already been shown in Figure 10.

The possible uncertainties of the numerical calculation of *dε*′∕*dlnω* from the observed ε′ data for sharp peaks brought us to use a well-defined alternative, the simple symmetric logarithmic difference Δε′ of ε′

(7.2) $$\begin{array}{l} \Delta \varepsilon^{\prime }\left(\omega \right)=\varepsilon^{\prime }\left(\omega ∕2\right)-\varepsilon^{\prime }\left(2\omega \right). \end{array}$$

If the input data are not available at a spacing of h = 2 then we can take more generally

(7.2.1) $$\begin{array}{l} \Delta_{h}\varepsilon^{\prime }\left(\omega \right)=\varepsilon^{\prime }\left(\omega ∕h\right)-\varepsilon^{\prime }\left(h\omega \right) \end{array}$$

**Figure 16:**
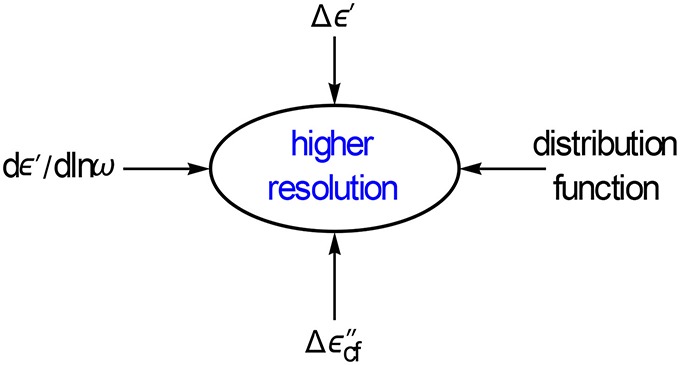
**We will show that additional functions like the logarithmic difference of Δε′ and the conduction free ε″ difference Δε″_*cf*_ will increase the resolution**. Interestingly, the handy Δε′ performs almost as good as *dε*′∕*dlnω*.

As depicted in Figure 16, we can also opt for logarithmic differences of ε″. Those differences should show like Δε′ a peak for each relaxation time present (when ω_*m*_ = 1/τ). Another prerequisite is that they should be conduction free, so that they cancel any possible contribution from ohmic conduction in the experimental ε″ data. This gives us two options an asymmetric one with two terms and a symmetric one with three terms

(7.3) $$\begin{array}{lll} \Delta \varepsilon_{fc}^{\prime \prime }\left(\omega \right) & = & -\varepsilon^{\prime \prime }\left(\omega ∕2\right)+2\varepsilon^{\prime \prime }\left(\omega \right) \end{array}$$

(7.4) $$\begin{array}{lll} \Delta \varepsilon_{cf}^{\prime \prime }\left(\omega \right) & = & -2\varepsilon^{\prime \prime }\left(\omega ∕2\right)+5\varepsilon^{\prime \prime }\left(\omega \right)-2\varepsilon^{\prime \prime }\left(2\omega \right). \end{array}$$

Here too we can use a spacing different from *h* = 2

(7.3.1) $$\begin{array}{lll} \Delta_{h}\varepsilon_{fc}^{\prime \prime }\left(\omega \right) & = & -\varepsilon^{\prime \prime }\left(\omega ∕h\right)+h\varepsilon^{\prime \prime }\left(\omega \right) \end{array}$$

(7.4.1) $$\begin{array}{lll} \Delta_{h}\varepsilon_{cf}^{\prime \prime }\left(\omega \right) & = & -h\varepsilon^{\prime \prime }\left(\omega ∕h\right)+\left(h^{2}+1\right)\varepsilon^{\prime \prime }\left(\omega \right)-h\varepsilon^{\prime \prime }\left(h\omega \right). \end{array}$$

It goes without saying that in the data analysis we should take h the same in all parts of the l.s.q. minimization. Hence, we should choose the same h in the sampling of the experimental data and in the theoretical model representation of Δε′ and Δε″. In order to simplify the minimization we best model Δε′ and Δε″ by taking:

(7.2.2) $$\begin{array}{lll} \Delta_{h}\varepsilon^{\prime }\left(\omega \right) & = & ℜ\left[\varepsilon^{*}\left(\omega ∕h\right)-\varepsilon^{*}\left(h\omega \right)\right] \end{array}$$

(7.3.2) $$\begin{array}{lll} \Delta_{h}\varepsilon_{fc}^{\prime \prime }\left(\omega \right) & = & -ℑ\left[-\varepsilon^{*}\left(\omega ∕h\right)+h\varepsilon^{*}\left(\omega \right)\right] \end{array}$$

(7.4.2) $$\begin{array}{l} \Delta_{h}{\varepsilon^{\prime \prime }}_{cf}(\omega )=-ℑ[-h\varepsilon^{*}(\omega /h)+(h^{2}+1)\;\varepsilon^{*}(\omega )\\ -h\varepsilon^{*}(h\omega )]. \end{array}$$

The reason being that the analytical expression for ε^*^ is often much simpler than that for ε′ and ε″. By using Equations (7.2.2–7.4.2) we let the l.s.q. routine compute the real and imaginary part of the differences, which is the most efficient way.

**Figure 17:**
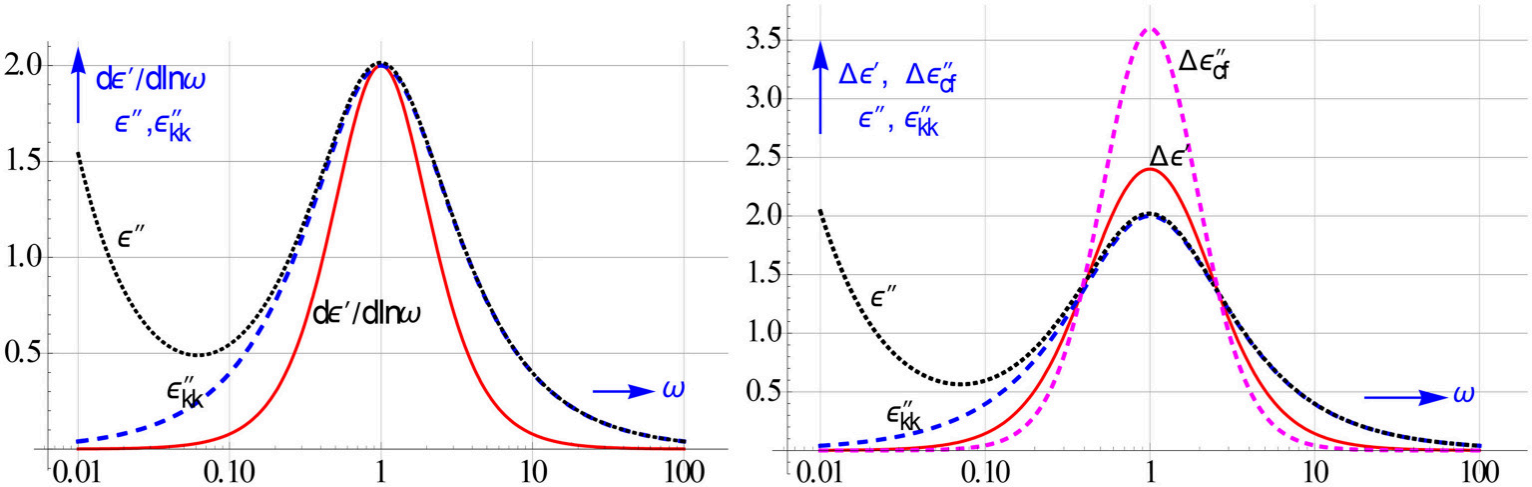
**On the left peak shape of *dε*′∕*dlnω* compared to that of ε″**. On the right the peaks of the logarithmic differences Δε′ and Δε″_*cf*_, in particular the latter is much spikier than ε″_*kk*_. The curves are given for a Debye relaxation and *h* = 2. The rise in ε″ in the low ω region is caused by ohmic conduction, it clearly lessens the resolution.

The increase in resolution by using the ε′ and ε″ differences is shown in Figure 17. Their peaks are like that of *dε*′∕*dlnω* much narrower than that of ε″ or ε″_*kk*_ for a Debye relaxation. However, they can be calculated much easier, while like *dε*′∕*dlnω* they have the added advantage that they too remove the conduction. It is further gratifying to notice that Δε′ enhances the resolution virtually as good as *dε*′∕*dlnω*.

**Figure 18:**
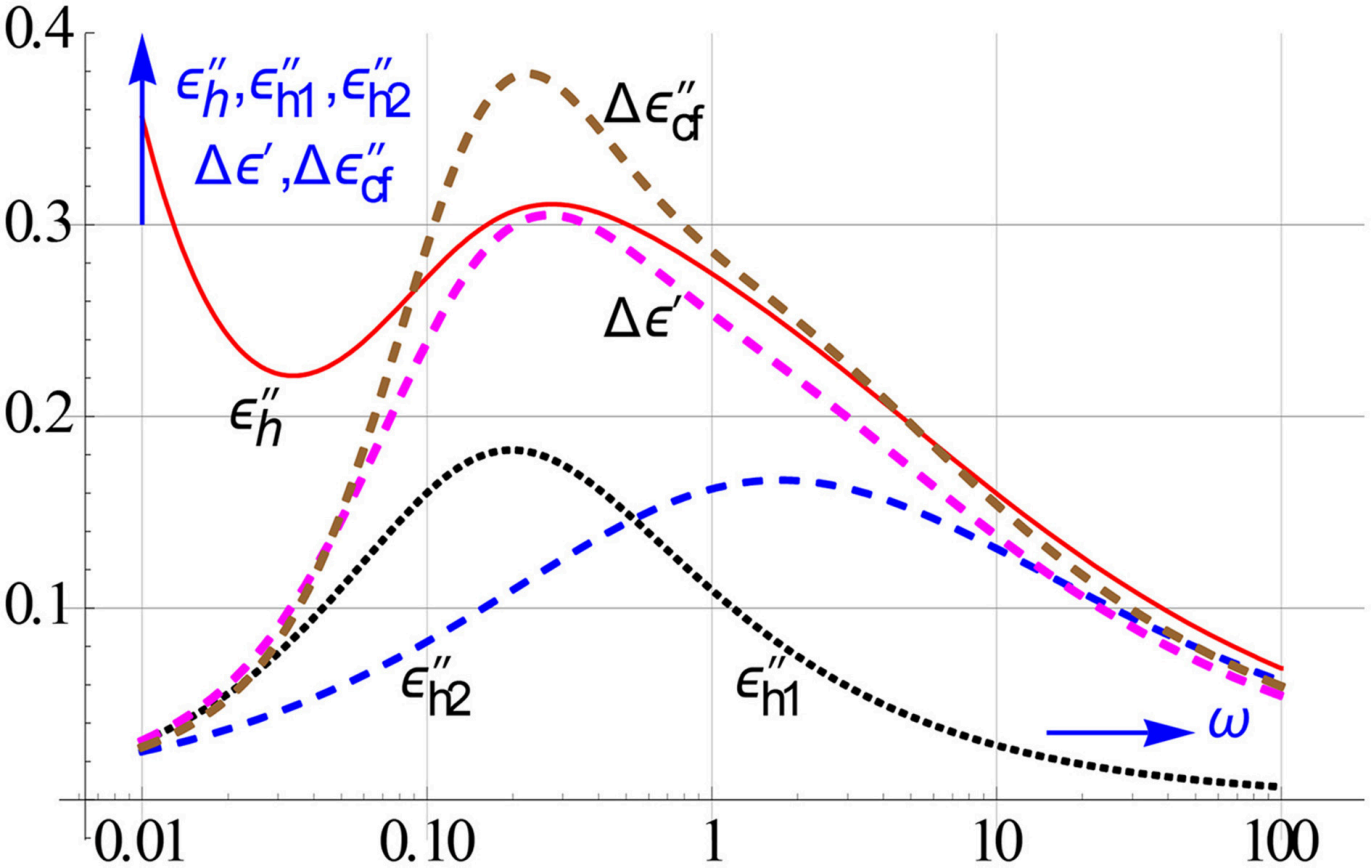
**Another illustration that Δε′ and Δε″_*cf*_ increase the resolution of dielectric spectroscopy**. We simulated the different spectra of 2 nearby HN relaxations, which fuse together into a single ε″_*h*_ peak. By contrast, the new differences do hint, even for these very close by relaxations, to the presence of 2 relaxations. This is in part due to the absence of the conduction loss in Δε′ and Δε″_*cf*_.

An enhanced resolution becomes of course more acute if the relaxation processes tend to overlap as often occurs in colloidal systems. Such a merger is also the case for the 2 HN relaxations considered in Figure 15. In Figure 18 we compare the ensuing spectra of Δε′ and Δε″_*cf*_ of these associated HN relaxations with the traditionally used spectrum of ε″_*h*_, which at low frequencies also contains the additional conduction loss. The 2 underlying HN relaxations can in particularly be conceived in the Δε″_*cf*_ spectrum. The distinction in the Δε′ spectrum is less, but better than in the ε″-curve, which merely shows one united peak.

We can next make use of Δε″_*cf*_ to get an approximation for *dε*′∕*dlnω* from a frame of 5 ε″ values via the cot operator that is conduction free. We warned that this was not the case with the frame of Equation (4.13).

We recall from Section Computing Conversion Frames with Symbolic Differential Operators.

(7.5) $$\begin{array}{l} \frac{d\varepsilon^{\prime }}{d\ln\;\omega }=D_{l}\cot\left(\pi D_{l}∕2\right)\left[\varepsilon^{\prime \prime }\left(\omega \right)\right]=\frac{\ln\;E_{l}}{\ln\;h}\cot\left(\frac{\pi \ln\;E_{l}}{2\ln\;h}\right)\left[\varepsilon^{\prime \prime }\left(\omega \right)\right]. \end{array}$$

We further have for Δε″_*cf*_

(7.6) $$\begin{array}{l} \Delta \varepsilon_{cf}^{\prime \prime }\left(\omega \right)=\left(-2∕E_{l}+5-2E_{l}\right)\varepsilon^{\prime \prime }\left(\omega \right). \end{array}$$

By replacing ε″(ω) by Δε″_*cf*_ in Equation (7.4) we get accordingly

(7.7) $$\begin{array}{l} \frac{d\varepsilon^{\prime }}{d\ln\;\omega }=\frac{\ln\;E_{l}}{\left(-2∕E_{l}+5+2E_{l}\right)\ln\;h}\cot\left(\frac{\pi \ln\;E_{l}}{2\ln\;h}\right)\left[\Delta \varepsilon_{cf}^{\prime \prime }\left(\omega \right)\right]. \end{array}$$

By series matching the r.h.s of Equation (7.7) for one term and three terms in E_*l*_ we get as coefficients: {0.63662} and {0.183437, 0.269745, 0.183437}. We combined these coefficients into one set so that the optimal approximation for *dε*′∕*dlnω* of a Debye relaxation was achieved. This gives for the best coefficients of the conversion of Δε″_*cf*_ from Equation (7.4) at respectively ω/2,ω and 2 ω to *dε*′∕*dlnω*: {0.0984351, 0.43975, 0.0984351}.

We can now turn back directly to the measured ε″-data. This results in the following 5 term frame for the conduction free conversion of the *observed* ε″-data into the logarithmic derivative of ε′

(7.8) $$\begin{array}{l} d\varepsilon^{\prime }∕d\ln\;\omega ≃\sum_{k=-2}^{2}e_{fk}\varepsilon^{\prime \prime }\left(2^{k}\omega \right), \end{array}$$

with e_*fk*_ = {−0.19687, −0.38732, 1.80501, −0.38732, −0.19687}.

**Figure 19:**
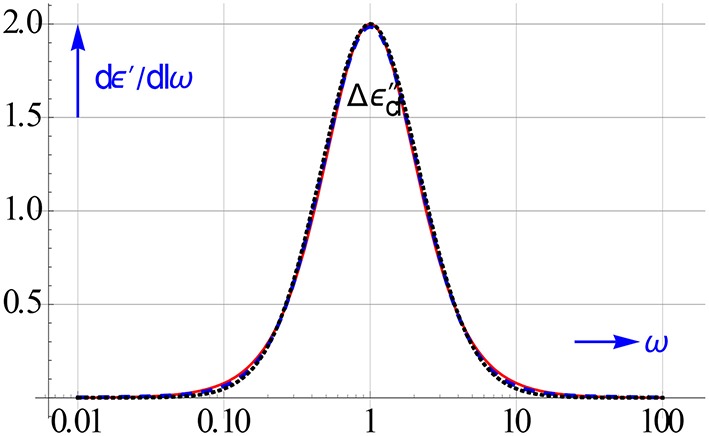
**The KK conversion allows the calculation of *dε*′∕*dlnω* from ε″ data**. The figure compares the plain, direct ε″ conversion of Equation (4.13) and the special one of Equation (7.8), by taking ε″ data of a Debye relaxation polluted by conduction.

The accuracy of the special conduction free conversion of ε″ to *dε*′∕*dlnω* for a Debye relaxation can be seen in Figure 19. It behaves about equally well as the unconstrained conversion of Equation (4.13). However, it performs slightly less at the wings than the mixed conversion of ε′ and ε″ of Equation (3.10), which is also conduction free.

## Enhancing the resolution by calculating simple approximations to the distribution function

If several concurrent relaxation processes are active, as is often the case in colloidal systems, then we can sort out the entire relaxation behavior comprehensively with a distribution function in relaxation times.

A Debye relaxation appears in such a distribution as a line spectrum. This is clear if we substitute a δ function in Equation (2.1) we then get

(8.1) $$\begin{array}{l} \varepsilon^{*}\left(\omega \right)=\varepsilon_{\infty }+\frac{\Delta \varepsilon }{1+i\omega \tau }. \end{array}$$

This shows that the distribution function offers us the highest resolution possible. A powerful method to obtain the distribution from a complex function like ε^*^ was proposed as long ago as 1894 by Stieltjes in his correspondence with Hermite about his pioneering work on continued fractions. His inversion relies on inserting for w → i/τ. This transforms the kernel in Equation (3.2) into a singularity and leads owing to Cauchy's integral theorem to

(8.2) $$\begin{array}{l} g\left(\ln\;\tau \right)=\tau f\left(\tau \right)=-\frac{1}{\pi }\;ℑ\left[\varepsilon^{*}\left(i∕\tau \right)\right], \end{array}$$

where g(lnτ) is the so-called logarithmic distribution function τf(τ).

In view of Equation (8.2) we can now take up the complex rational fractional power approximation of Equation (6.2) and simply find

(8.3) $$g(\ln\tau )=-\frac{1}{\pi }ℑ\frac{\sum_{k=0}^{n}a_{k}{\left(-1/\tau \right)}^{ck}}{1+{\sum_{k=1}^{n+1}b_{k}(-1/\tau )}^{ck}}.$$

We can further make use of Equation (4.2) and the fact that lni = iπ/2 to derive in a simple way the symbolic differential operators for the inversion of ε′ and ε″. By expressing ε^*^(iω) in ε′ and ε″ we get:

(8.4) $$\begin{array}{l} \begin{array}{c} \varepsilon^{*}\left(i\omega \right)=e^{D_{l}\ln\;i}\varepsilon^{*}\left(\omega \right)=\left[\cos\left(\pi D_{l}∕2\right)+i\sin\left(\pi D_{l}∕2\right)\right]\varepsilon^{*}\left(\omega \right)=\\ \left[\cos\left(\pi D_{l}∕2\right)+i\sin\left(\pi D_{l}∕2\right)\right]\left[\varepsilon^{\prime }\left(\omega \right)-i\varepsilon^{\prime \prime }\left(\omega \right)\right] \end{array} \end{array}$$

This gives in view of Equation (8.2) for the symbolic inversion via ε′ and ε″

(8.5) $$\begin{array}{lll} \sin\left(\pi D_{l}∕2\right)\left[\varepsilon^{\prime }\left(\omega \right)\right] & = & -\left(\pi ∕2\right)g\left(\ln\;\tau \right)\\ \cos\left(\pi D_{l}∕2\right)\left[\varepsilon^{\prime \prime }\left(\omega \right)\right] & = & \pi ∕2g\left(\ln\;\tau \right). \end{array}$$

It may be worthwhile to point out that the sin and cos inversion operator and the tan and cot KK operator are compatible. In fact by dividing the sin and cos operations in Equation (8.5) for g(lnτ) we just get our KK tan operator of Equation (4.9).

By using the product or Mittag-Leffler approximations in D_*l*_ for the sin and cos operator, we can show that crude approximations to g(lnτ) can be obtained from:

(8.6) $$\begin{array}{l} \frac{d\varepsilon^{\prime }}{d\ln\;\omega },\varepsilon^{\prime \prime }+\frac{d\varepsilon^{\prime \prime }}{d\ln\;\omega },\varepsilon^{\prime \prime }-\frac{d^{2}\varepsilon^{\prime \prime }}{d\ln{\;}^{2}\omega }, \end{array}$$

with ω → 1/τ (see e.g., Tschoegl, 1989). If we discretize the derivatives in Equation (8.6), then we obtain as differences

(8.7) $$\begin{array}{lll} \Delta \varepsilon^{\prime }\left(\omega \right) & = & \varepsilon^{\prime }\left(\omega ∕2\right)-\varepsilon^{\prime }\left(2\omega \right)\;\Delta \varepsilon_{fc}^{\prime \prime }\left(\omega \right)=-\varepsilon^{\prime \prime }\left(\omega ∕2\right)+2\varepsilon^{\prime \prime }\left(\omega \right)\\ \Delta \varepsilon_{cf}^{\prime \prime }\left(\omega \right) & = & -2\varepsilon^{\prime \prime }\left(\omega ∕2\right)+5\varepsilon^{\prime \prime }\left(\omega \right)-2\varepsilon^{\prime \prime }\left(2\omega \right). \end{array}$$

We have manipulated the discretization of the derivatives of ε″ somewhat so that with just 2 and 3 terms full nullification for ohmic conduction was achieved.

We used these logarithmic differences already in Section Simple Routes to a Higher Spectral Resolution: Logarithmic Derivatives or Differences of ε′ and ε″. It is now no longer surprising that we found them to promote the spectral resolution, as we have already demonstrated in Figures 17, 18. The mix of ε″+ *dε*″/dlnω was proposed by Kaatze (2003), as a means to remove the ohmic conduction. In fact ε″(ω)−*d*^2^ε″∕*d*ln^2^ω is a better choice, not only because it is symmetric, but also because it has a higher resolution. Obviously, our differences of ε″ are much easier calculated than the combination of ε″ with its derivatives, while their performance almost matches that of the differential expressions.

**Figure 20:**
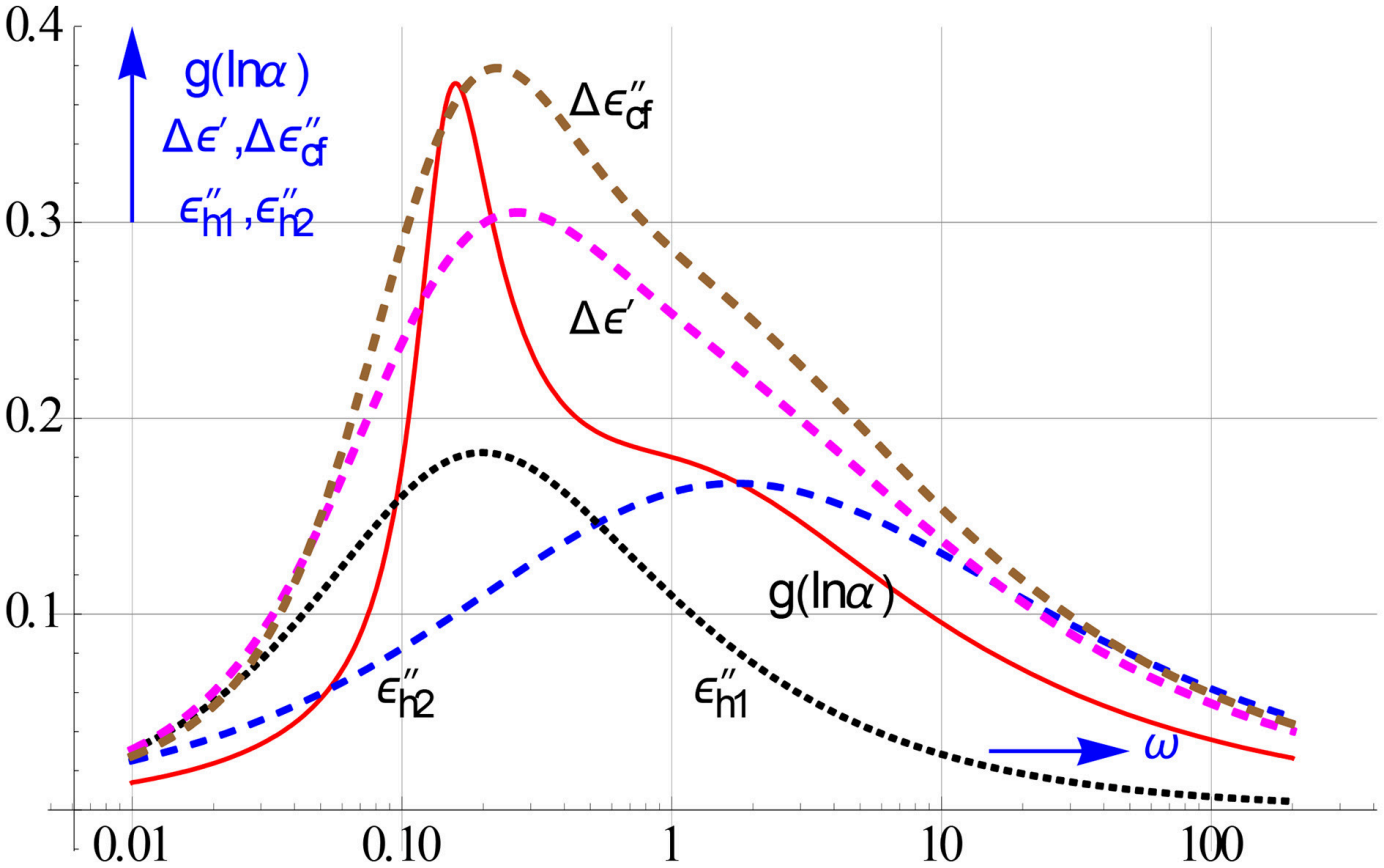
**Evidence of the strong resolution power of the distribution function**. Using Equation (8.3) we computed the distribution from a rational all-in-1 modeling to ε′and ε″of the 2 overlapping HN relaxations employed before in Figures 15, 18. The underpinning relaxations can also be distinguished in the spectra of the differences Δε′and Δε″_*cf*_, this is in particular true for the latter.

The performance of the difference approximations for decomposing the overlapping HN relaxations used earlier in Figure 15 compared to that of the distribution obtained with Equation (8.3) is shown in Figure 20. Since Stieltjes inversion is based on ω → i/τ, we have rather plotted g(lnα), where α = 1/τ. In this way we can use the same scale for all spectra.

Clearly the exact complex Stieltjes inversion performs best. The curve of the distribution function reveals convincingly the 2 HN relaxations present. The next best resolution is provided by Δε″_*cf*_. The simple difference approximations on the other hand have the advantage that they can be applied directly to the measured data, and thus readily provide straight insight via plots in the relaxation behavior under study. The location of the peaks in the plots supply good estimates for the various relaxation times that are active. These estimates, which often lie decades apart, can be used as initial values in the final mathematical modeling.

## Improving the resolution by all-in-1 modeling of the real and imaginary data

It is often illuminating if overlapping relaxations can be separated visually in plots. We showed in Section Enhancing the Resolution by Calculating Simple Approximations to the Distribution Function that this can be achieved by plotting approximations to the distribution function like Δε′ and Δε″_*cf*_ or even more so by plotting the distribution function itself by invoking the complex Stieltjes inversion.

However, we should realize that appropriate *fitting* offers a much more powerful tool to separate nearby relaxations. The common approach is to model a multiple relaxation with a sum of HN functions. By modifying Equation (6.1) to a sum, we have

(9.1) $$\begin{array}{l} \varepsilon_{hn}^{*}\left(\omega \right)=\varepsilon_{\infty }+\sum_{k=1}^{n}\frac{\Delta \varepsilon_{k}}{{\left[1+{\left(i\omega \tau_{k}\right)}^{a_{k}}\right]}^{b_{k}}}-\frac{i\gamma }{\varepsilon_{o}\omega }. \end{array}$$

Even a limited sum of 2 HN functions and one conduction term leads to no less than 10 unknowns. This hampers a proper mathematical separation of the 2 processes, the more so because the l.s.q. minimization is a nonlinear one.

The usual approach is to minimize the deviations between the measured and the HN-model values of ε′ and ε″ in one sum as follows

(9.2) $$\begin{array}{l} \sum_{k=1}^{n}{\left[\varepsilon^{\prime }\left(\omega_{k}\right)-\varepsilon_{hn}^{\prime }\left(\omega_{k}\right)\right]}^{2}+{\left[\varepsilon^{\prime \prime }\left(\omega_{k}\right)-\varepsilon_{hn}^{\prime \prime }\left(\omega_{k}\right)\right]}^{2}=\min. \end{array}$$

However, a better choice is to split the sum in two and minimize

(9.3) $$\begin{array}{l} \sum_{k=1}^{n}{\left[\varepsilon^{\prime }\left(\omega_{k}\right)-\varepsilon_{hn}^{\prime }\left(\omega_{k}\right)\right]}^{2}=\min,\overset{n}{\underset{k=1}{\sum }}{\left[\varepsilon^{\prime \prime }\left(\omega_{k}\right)-\varepsilon_{hn}^{\prime \prime }\left(\omega_{k}\right)\right]}^{2}=\min. \end{array}$$

**Figure 21:**
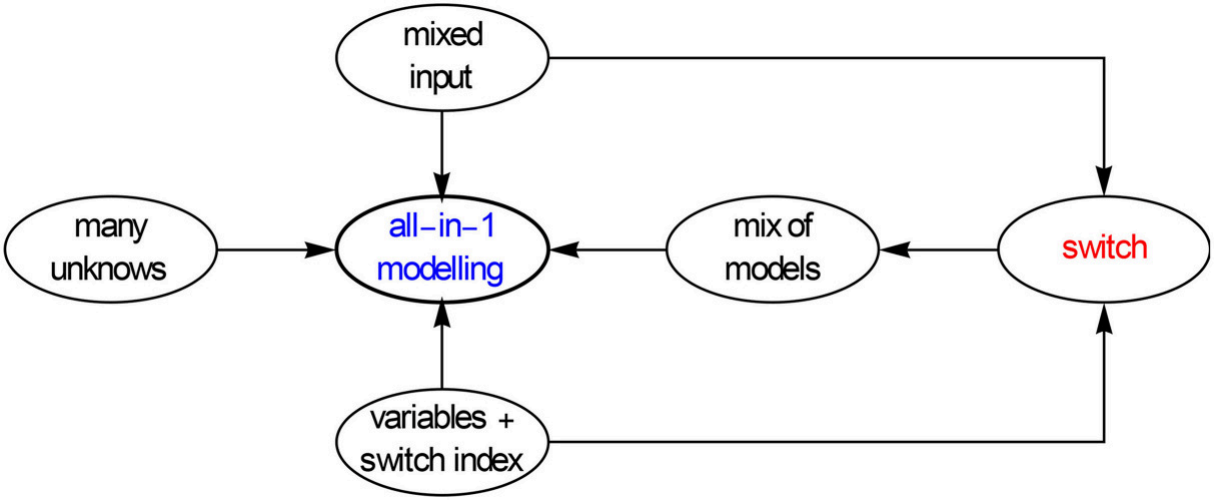
**Scheme of the all-in-1 or apart-together l.s.q. modeling**. The strategy is to link the mix of input data to the mix of models via an extra dummy variable, in fact an index, that acts as a switch and thus takes care of the correct link.

This apart-together or all-in-1 fitting can be achieved by making use of a mathematical switch, which combines the proper data, say ε′, with the proper model i.e., $\varepsilon_{hn}^{\prime }=ℜ\left[\varepsilon_{hn}^{*}\right]$, etc. Such a coupled minimization assures that the HN parameters in ε′_*hn*_ and ε″_*hn*_ are not allowed to differ, which clearly is a necessity. Figure 21 outlines the basics of the all-in-1modeling.

Most commercial software, such as Maple, Mathematica, Matlab, have a nonlinear l.s.q. procedure. We should preferably use the ones with the Levenberg-Marquardt routine built-in. They should also allow for the minimization of more than one variable. A typical example is the routine FindFit of Mathematica, in which the switching can be implemented with several commands. One of the switches we use is based on the conditional if statement, which can be applied in other software as well. Another one is e.g., Kronecker's delta.

FindFit has the following structure FindFit [input data, model, unknowns, variables]. It gives the values of the unknowns as output. Assuming that we do the all-in-1 modeling for ε′ and ε″, with two HN's and one conduction term, we have

(9.4) $$\begin{array}{l} \varepsilon_{hn}^{*}\left(\omega \right)=\varepsilon_{\infty }+\overset{2}{\underset{k=1}{\sum }}\frac{\Delta \varepsilon_{k}}{{\left[1+{\left(i\omega \tau_{k}\right)}^{a_{k}}\right]}^{b_{k}}}-\frac{i\gamma }{\varepsilon_{o}\omega }. \end{array}$$

We should then activate FindFit as follows,

input data: Join[Table[{ω_*k*_, 1, ε′(ω_*k*_)}, {k, n}], Table[{ω_*k*_, 2, ε″(ω_*k*_)}, {k, n}].

model: If[i = 1, ε′_*hn*_(ω), 0] + If[i = 2, ε″_*hn*_(ω), 0].

unknowns: ε_∞_, Δε_1_, a_1_, b_1_, τ_1_, Δε_2_, a_2_, b_2_, τ_2_ and γ.

variables: ω, i.

We have thus added to ω an extra variable i. This is an index that controls the if switch and makes the proper choice between the input data and the ε′ and ε″ models, for i = 1 it selects the ε′ data and links it to the ε′-model, while for i = 2 it selects and combines the ε″-data and the ε″-model. This is the reason that the input data comprise along with ω_*k*_ and the function values for ε′(ω_*k*_) and ε″(ω_*k*_), the index i as 2nd variable. In doing so we have transformed the common univariate minimization in ω, into a quasi-bivariate (or two-dimensional) minimization in ω and i. Although ε′_*hn*_ and ε″_*hn*_ can be written out analytically, we better model them as suggested earlier with

(9.5) $$\begin{array}{l} \varepsilon_{hn}^{\prime }\left(\omega \right)=ℜ\left[\varepsilon_{hn}^{*}\left(\omega \right)\right]\text{ and }\varepsilon_{hn}^{\prime \prime }\left(\omega \right)=-ℑ\left[\varepsilon_{hn}^{*}\left(\omega \right)\right], \end{array}$$

in order to keep the minimization simple.

The all-in-1 modeling can of course be extended to more input data and more model functions. In particular data and models for Δε′ and Δε″ are attractive, because they have intrinsically a better resolution power. They are therefore more powerful in separating well the genuine colloid relaxations from that of the electrode polarization. Both have the additional advantage over ε″ that they are not affected by ohmic conduction. Furthermore, the models for both are simple for e.g., Δε′ we frankly have

(9.6) $$\begin{array}{l} \Delta \varepsilon^{\prime }\left(\omega \right)=ℜ\left[\varepsilon_{hn}^{*}\left(\omega ∕2\right)-\varepsilon_{hn}^{*}\left(2\omega \right)\right]. \end{array}$$

**Figure 22:**
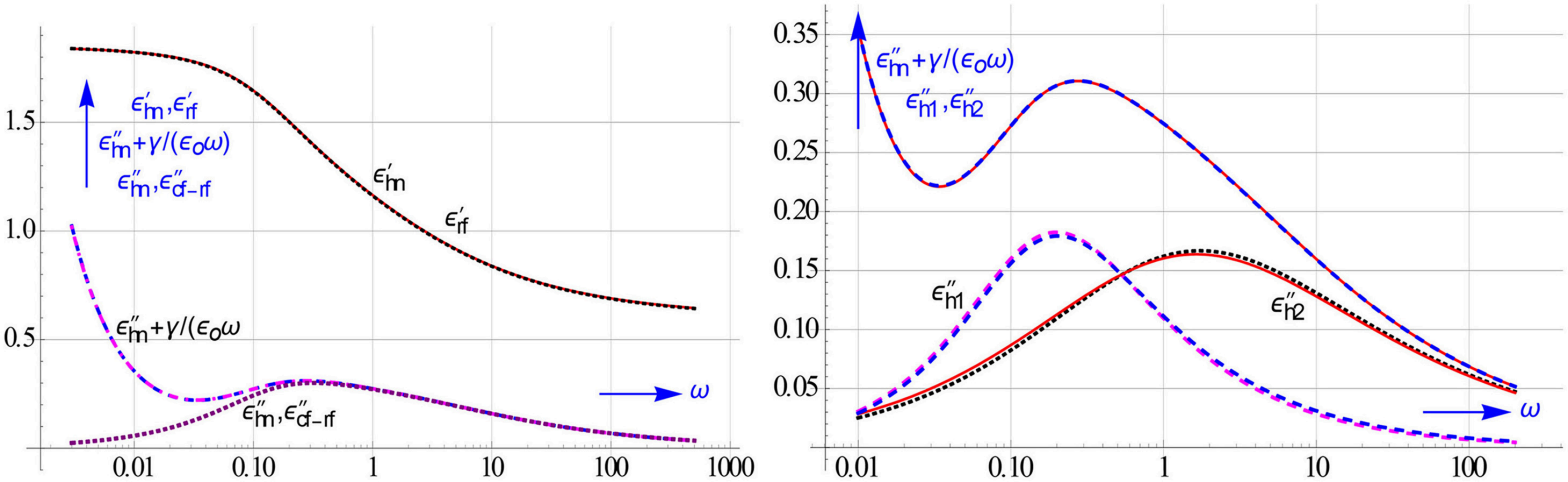
**Decomposing 2 overlapping HN relaxations with all-in-1 modeling**. Despite the fact that the 2 HN relaxations almost blend into one we could recover, as the results on the right show, each of the two genuine HN relaxations comfortably. Even though the simulated data were disturbed with random errors of ±1%.

Figure 22 illustrates what can be achieved with an all-in-1 modeling of 2 overlapping HN relaxations, using Equation (9.4). Visually it seems that we are only dealing with 1 relaxation. However, by using not only ε′ and ε″, but Δε′ and Δε″_*cf*_ as input as well, we could unravel the 2 underlying HN's nicely, despite the fact that we have contaminated the data in this simulation with random relative errors of ±1%. We can see on the r.h.s. of Figure 22 that the recalculated ε″_*h*1_ and ε″_*h*2_ relaxation peaks closely resemble the original exact curves. The data were generated for: ε_∞_ = 0.6, Δε_1_ = 0.75, a_1_ = 0.6, b_1_ = 0.7, τ_1_ = 1, Δε_2_ = 0.5, a_2_ = 0.9, b_2_ = 0.7, τ_2_ = 7, and γ = 0.003, with the all-in-1 modeling we recovered these values as: ε_∞_ = 0.60, Δε_1_ = 0.75, a_1_ = 0.58, b_1_ = 0.74,τ_1_ = 1, Δε_2_ = 0.50, a_2_ = 0.91, b_2_ = 0.66, τ_2_ = 7.22, and γ = 0.003.

The HN function is just an empirical model function. A much better way to characterize the various dielectric properties of colloidal systems is by making use of the complex dipolar coefficient β^*^.

This function is defined as follows, if we have a mixture of two components, say of spherical particles dispersed in a medium, then we can model according to Maxwell and Wagner the complex permittivity of this mixture $\varepsilon_{m}^{*}$ by Grosse (2002) and Steeman and van Turnhout (2002)

(9.7) $$\begin{array}{l} \frac{\varepsilon_{m}^{*}\left(\omega \right)-\varepsilon_{b}^{*}\left(\omega \right)}{\varepsilon_{m}^{*}\left(\omega \right)+\varepsilon_{b}^{*}\left(\omega \right)}=\phi \frac{\varepsilon_{p}^{*}\left(\omega \right)-\varepsilon_{b}^{*}\left(\omega \right)}{\varepsilon_{p}^{*}\left(\omega \right)+\varepsilon_{b}^{*}\left(\omega \right)}=\phi \beta^{*}\left(\omega \right), \end{array}$$

where $\varepsilon_{b}^{*}$ is the complex permittivity of the bulk, $\varepsilon_{p}^{*}$ that of the particles, ϕ their volume fraction and β^*^(ω) the complex dipolar coefficient. We can rewrite Equation (9.6) to an explicit expression for $\varepsilon_{m}^{*}$

(9.8) $$\begin{array}{l} \varepsilon_{m}^{*}\left(\omega \right)=\varepsilon_{b}^{*}\left(\omega \right)\frac{1+2\phi \beta^{*}\left(\omega \right)}{1-\phi \beta^{*}\left(\omega \right)}. \end{array}$$

In colloidal suspensions the bulk consists of the suspending electrolyte so ε${}_{b}^{*}=$ ε${}_{e}^{*}$. We see from Equation (9.6) that β^*^(ω) is determined by $\varepsilon_{p}^{*}$ and $\varepsilon_{e}^{*}$. The electrolyte is full of ions which are pushed by the alternating electric field to and fro the electrodes and the (insulating) particles. The ionic motion is however in part also controlled by diffusion. Near the electrodes the ions form electric double layers, which give rise to a strong EP. The motion of the ionic clouds near the particles give rise to special relaxations. The permittivity of the particles ε${}_{p}^{*}$ therefore is not simply due to the dipole relaxations and possible ohmic conduction. We should further realize that the particles may carry a charge (this depends on their ζ potential).

All in all it is quite a challenge to account for the many processes possible. Expressions for β^*^(ω) have e.g., been given by Grosse (2002) and Chassagne and Bedeaux (2008). The β^*^-model from Chassagne and Bedeaux (2008) was not fully explicit. Fortunately, it has been possible to achieve this lately, further details are given in Chassagne et al. (submitted).

Replacing $\varepsilon_{m}^{*}$ by $\varepsilon_{s}^{*}$, the permittivity of the suspension, and $\varepsilon_{b}^{*}$ by $\varepsilon_{e}^{*}$, the permittivity of the electrolyte, we get

(9.9) $$\begin{array}{l} \varepsilon_{s}^{*}\left(\omega \right)=\varepsilon_{e}^{*}\left(\omega \right)\frac{1+2\phi \beta^{*}\left(\omega \right)}{1-\phi \beta^{*}\left(\omega \right)}≃\varepsilon_{e}^{*}\left(\omega \right)\left[1+3\phi \beta^{*}\left(\omega \right)\right], \end{array}$$

where $\varepsilon_{e}^{*}\left(\omega \right)=\varepsilon_{w}-i\varepsilon_{w}∕\left(\omega \tau_{e}\right)$, with τ_*e*_ = ε_*o*_ε_*w*_∕γ_*e*_. However, if we measure the capacity of a cell with a colloidal suspension, then it includes a large contribution caused by the EP. This huge contribution manifests itself at low frequencies, because the ions move rather slowly.

**Figure 23:**
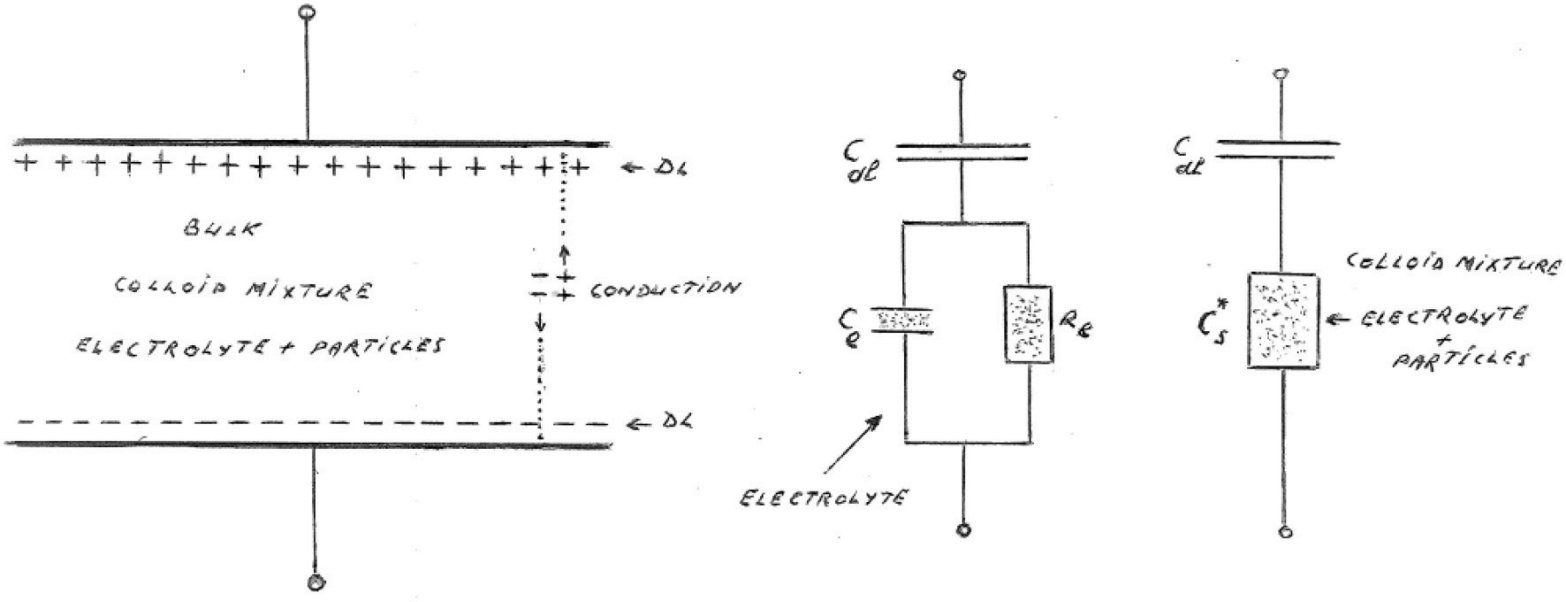
**Series model for the total complex capacitance of a colloidal suspension in a measuring cell**. The model on the right shows the true complex capacitance of the suspension $C_{s}^{*}$ in series with the DL capacitance, which is responsible for the EP. The circuit in the middle shows the series model for the electrolyte without the colloidal particles. The model on the left depicts the ion layers near the electrodes, when the top electrode is negative and the bottom one positive. We have also pointed to a continuous flow of ions, This ongoing flow is only possible if the electrodes are partially blocking. In our theoretical analysis, we assume that the electrodes are fully blocking, the EP is then at its strongest.

The usual way to account for EP is to use a series circuit model as depicted in Figure 23. This consists of a large capacitor filled with electrolyte devoid of its ions. This represents the part formed by the two electric double layers near the electrodes. The DL capacitor is tied up in series with the complex bulk capacitor of the suspension, which is a mixture of the electrolyte and the colloidal particles. This leads to

(9.10) $$\begin{array}{l} d∕\varepsilon_{cs}^{*}\left(\omega \right)=2d_{l}∕\varepsilon_{w}+\left(d-2d_{l}\right)∕\varepsilon_{s}^{*}\left(\omega \right)≃\;2d_{l}∕\varepsilon_{w}+d∕\varepsilon_{s}^{*}\left(\omega \right), \end{array}$$

where $\varepsilon_{cs}^{*}$ denotes the complex permittivity of the suspension as measured with the cell, ε_*w*_ the (real) permittivity of water and $\varepsilon_{s}^{*}$ the true complex permittivity of the suspension. We may neglect 2d_*l*_ against d in Equation (9.9), because the Debye length d_*l*_ is extremely small for aqueous systems. Note that d_*l*_ is known analytically, and can be calculated using handbook values e.g., from Weast (1987).

To simplify matters we suppose that the electrodes in the series model are totally blocking any charge exchange. We can generalize the model to partial blocking, the EP then becomes less dominant, but this will not be pursued here (see e.g., Coelho, 1983). Although it should be stressed that it is only for partial blocking that we will observe ohmic conduction losses.

We rather like to illustrate the use of Equations (9.9) and (9.10) in all-in-1 modeling. Obviously, we are mainly, not to say only, interested in the true response of the colloidal particles free of EP. This can again be achieved by using Δε′ and $\Delta \varepsilon_{cf}^{{}^{\prime \prime }}$ as input. They should be calculated from the $\varepsilon_{cs}^{\prime }$ and $\varepsilon_{cs}^{\prime \prime }$ values measured. However, we can model Δε′ and $\Delta \varepsilon_{cf}^{{}^{\prime \prime }}$ by taking straightaway $\varepsilon_{s}^{*}$ from Equation (9.9). We need not to use the more complicated theoretical for Δε′ and $\Delta \varepsilon_{cf}^{{}^{\prime \prime }}$ from $\varepsilon_{cs}^{*}$ measured with the EP included. This simplification is allowed in the ω region where the EP has died out. This ω region becomes readily visible from the Δε′ and Δε${}_{cf}^{{}^{\prime \prime }}$ plots. So in the apart-together model with built-in switch we take:

$$\begin{array}{l} if\left[i=1,\Delta \varepsilon^{\prime }\left(\omega \right)=ℜ\left[\varepsilon_{s}^{*}\left(\omega ∕2\right)-\varepsilon_{s}^{*}\left(2\omega \right)\right],0\right]+\\ if\left[i=2,\Delta \varepsilon_{fc}^{\prime \prime }\left(\omega \right)=-ℑ\left[-\varepsilon_{s}^{*}\left(\omega ∕2\right)+2\varepsilon_{s}^{*}\left(\omega \right)\right],0\right] \end{array}$$

where $\varepsilon_{s}^{*}$ follows from Equation (9.9) wherein ε${}_{e}^{*}$ can be specified for most electrolytes using the conductivities given in a handbook like (Weast, 1987). Instead of $\Delta \varepsilon_{fc}^{\prime \prime }$ we can also use the symmetric difference Δε″_*cf*_. The basic parameters in β^*^ are the zeta potential and the particle radius a_*o*_ (see Chassagne et al., submitted). It are these important unknowns that will come out of the all-in-1 fit.

An unique aspect of the β^*^ function over the HN fit is that it allows the fitting of multiple relaxation processes with just *one* function, provided that it is explicit and that in the β^*^ model all underlying relaxations are properly accounted for Chassagne et al. (submitted).

**Figure 24:**
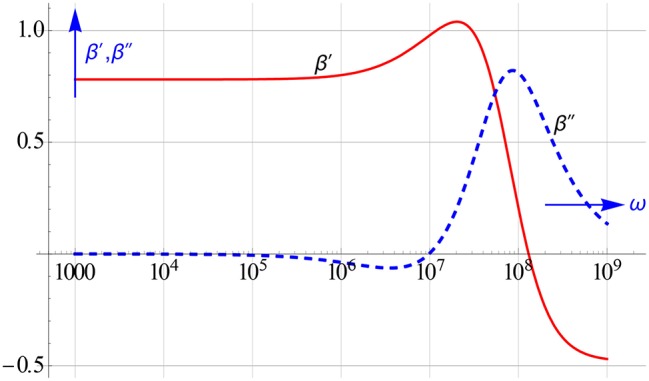
**The β′and β″ spectra for a colloidal mixture of 50 nm sized spherical particles with ε_*p*_ = 2 carrying a charge equivalent to a ζ potential of 4 suspended in an 1:1 electrolyte**.

The real and imaginary part of the dipolar coefficient of 50 nm particles with a zeta-potential of 4 dispersed in an 1:1 electrolyte is shown in Figure 24. We generated these curves by taking the same parameter values for the electrolyte as mentioned in Chassagne et al. (submitted) Interestingly, we could retrieve the given particle size and zeta-potential accurately by performing the all-in-1 fit as outlined above. The recalculated β′ and β″ curves coincide nicely with the exact ones across the whole ω range. We found that this remains so, if we include random errors in the input data.

The dipolar coefficient represented in Figure 24 actually gives rise to two distinct relaxation phenomena at high frequencies. These relaxations do not show up in the ε″ curve, because the ε″ loss is dominated and controlled by that of the electrolyte and so is very high. However, they do emerge brightly in the logarithmic differences of ε′ and ε″.

**Figure 25:**
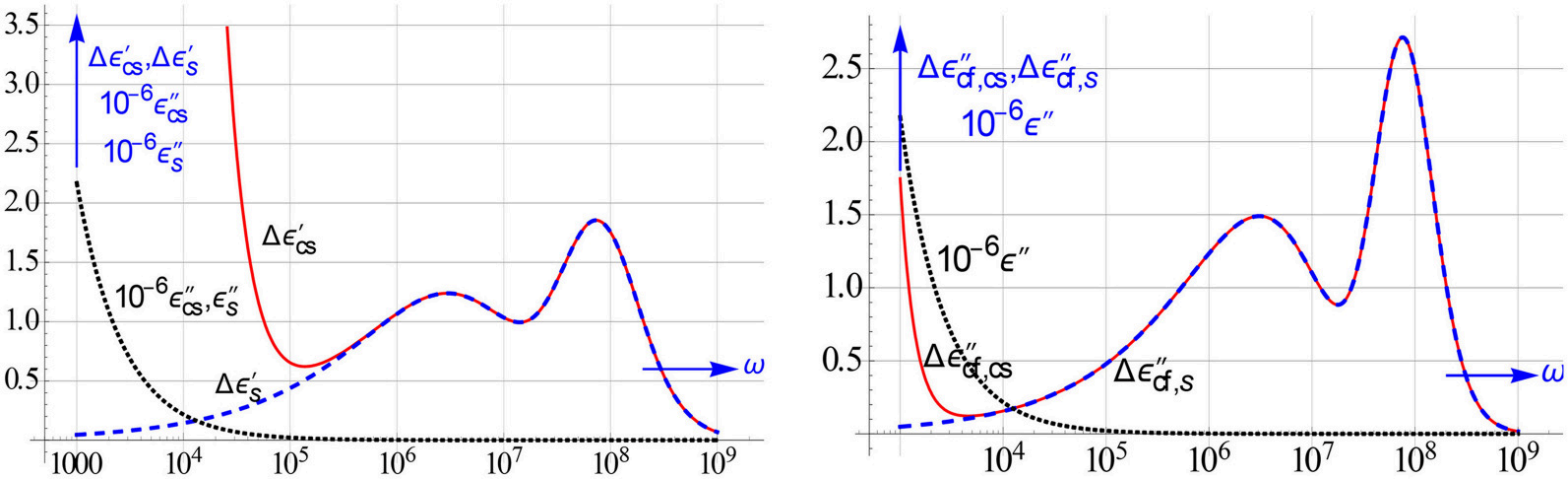
**Plots of Δε′, Δε${}_{\text{cf}}^{{}^{\prime \prime }}$, and ε″ for the measured data (labeled with subscript cs) and the true values of the suspension itself (labeled with s)**. The ε″ loss is very high, so we have rather plotted 10^−6^ε″. The data were generated with the β^*^ model of Figure 24. The ε″ curves do not reveal the factual colloidal relaxations present. By contrast the difference curves do so indisputably, in particular the Δε${}_{\text{cf}}^{{}^{\prime \prime }}$ curves exhibit two prominent peaks.

This is demonstrated in Figure 25, which shows on the left Δε′ and on the right $\Delta \varepsilon_{cf}^{{}^{\prime \prime }}$ for the same colloidal suspension with ζ = 4 and particle radius a_*o*_ = 25 nm. They unravel that such a suspension shows right after the EP relaxation, two clear relaxation peaks due to the particles. Both could be nicely fitted with one and the same β^*^ model using Δε′ and $\Delta \varepsilon_{cf}^{{}^{\prime \prime }}$. Not surprisingly, we could therefore ascertain the parameter values used in the simulated data quite accurately. It may be worth pointing out that the steep rise at low frequencies in Δε′_*cs*_ and Δε${}_{cf,cs}^{{}^{\prime \prime }}$, which quantities both derive from the measured data, is brought about by the EP in the measuring cell. Comparing the Δε′_*cs*_ and Δε${}_{cf,cs}^{{}^{\prime \prime }}$ curves we see that this rise starts earlier, i.e., at higher ω's, for $\Delta \varepsilon_{cs}^{\prime }$. This means that even in the differences of the measured cell data the genuine relaxation peaks come out quite reliably. This is specially apparent in the Δε${}_{cf,cs}^{{}^{\prime \prime }}$ spectrum. It is by virtue of this close overlap between Δε′_*s*_ vs. Δε′_*cs*_ and Δε${}_{cf,s}^{{}^{\prime \prime }}$ vs. Δε${}_{cf,cs}^{{}^{\prime \prime }}$ that the all-in-1 modeling is so successful.

Clearly, we would have found a very similar close agreement between the measured and true curves of the logarithmic derivative *dε*′∕*dlnω* beyond the EP region. The possibility to remove with a more advanced data analysis the EP-contribution was earlier established and explored by Jimenez et al. (2002). Instead of *dε*′∕*dlnω* we now rather advocate and prefer the use of Δε′ and $\Delta \varepsilon_{cf}^{{}^{\prime \prime }}$, because these well-defined quantities are much easier to calculate from the data and to model. It should be stressed that actually an analytical model for *dε*′/dlnω of Equation (9.9) cannot be given, because β^*^(ω) can only only be represented by a very complicated expression, cf. Chassagne et al. (submitted)^3^.

## Discussion

The use of the singular KK integrals for the interconversion between the real and imaginary part is still limited. They are mainly used in impedance spectroscopy to discover spurious effects in the data analysis caused by measuring errors (Barsoukov and Macdonald, 2005; Orazem and Tribollet, 2008).

By taking up the real part sufficiency based on the KK relations we have managed to convert ε′ into ε″ data. In this way conduction free ε″ losses can be calculated that allow us to uncover the genuine l.f. colloidal dispersions that otherwise remain hidden below strong conduction losses. This dissipative energy loss originates from the continuous flow of ions in the electrolyte toward the electrodes. It has nothing to do with the true relaxations of the colloidal particles.

We have strived for a fast and easy KK conversion. This was achieved by replacing the KK integrals by conversion frames, which consist of a window of coefficients by which a set of logarithmically spaced ε′ data are multiplied. By moving such frames step by step along all ε′ data we get a new set of ε″ fully cleaned from ohmic conduction. The cleaned ε″ data can only come from the genuine relaxation losses. The best method to derive the easy to use conversion frames is kernel matching. This versatile method can also provide panels for other conversions such as from ε″ to *dε*′∕*dlnω*. We took care to do the latter in such a way that this conversion remains unaffected by possible conduction in the observed ε″-data. This requires a special frame of logarithmically spaced ε″-data.

As other source to generate conversion frames we explored symbolic differential operators. They were found by replacing the conventional KK integration by differentiation. This option is possible because the KK integrals can be seen as logarithmic convolution integrals. We invoked the logarithmic shift operator E_*l*_ to expand the cot operator in an apt way in conversion frames for *dε*′∕*dlnω*. We further showed that this operator plays a key role in Zahner's software for the logarithmic form of the KK conversion (Schiller et al., 2001; Lasia, 2014).

Since the dielectric response of colloidal systems often involves several processes (Grosse, 2002; Chassagne and Bedeaux, 2008; Delgado et al., 2014; Chassagne et al., submitted), we paid also attention to an improvement of the resolution power for the entire dielectric spectrum. Major gains in this respect could be made by using derivatives or differences in ε′ and ε″. The logarithmic differences made up of a few terms are the easiest to use, while they perform almost as good as the derivatives. Our approach has been to use differences based on approximations to the distribution function.

The ensuing distribution in fact outperforms all other methods and produces the highest resolution possible. We calculated the distribution to a high accuracy by making use of Stieltjes's complex inversion. For this purpose we approximated ε′ and ε″ by a complex rational fractional power function. This model free function has the additional advantage that it also allows a direct interconversion between ε′ and ε″. A KK conversion is no longer needed.

A better resolution shows up visually in plots vs. frequency. An even higher resolution of nearby relaxations can be achieved by mathematical modeling. A common approach is to describe each relaxation process present in an empirical way by a Cole-Cole function or Havriliak-Negami function. The latter is the most general. A sum of a few HN functions contains a lot of adjustable parameters. This makes the nonlinear l.s.q. minimization a formidable task.

The accuracy of the parameter estimation and thus of the reliability of the resolution could be improved a lot by developing a joint “apart-together” minimization of the real and imaginary parts. We have called this all-in-1 modeling and incorporated it in Mathematica's l.s.q. one liner routine FindFit. The trick is that we have built in a mathematical switch that links the proper input data to the proper part of the model function. The switch is controlled by an index i that is included as dummy variable in FindFit, which transforms it from an univariate ω-minimization into a pseudo bivariate ω,i-fit.

We successfully retrieved with the all-in-1 modeling those parameters that are the most crucial in the dielectric response of colloidal suspensions, viz. the charge and size of the particles. We could recover these vital unknowns by using the explicit β-model discussed in Chassagne et al. (submitted) for the dipolar coefficient of a suspension.

The ohmic conduction is not the only disturbance that poses a problem to the characterization of colloidal systems with DS. The electrode polarization may be an even larger thread to the unraveling of the true nature of the l.f. dispersion. The elimination of this hindrance by mere data fitting is touched upon in Section Improving the resolution by all-in-1 modeling of the real and imaginary data. Its removal is recently dealt with to a greater extent in Chassagne et al. (submitted) and van Turnhout et al. (2016), see also Ishai et al. (2013).

The KK relations can be applied to many areas (Peiponen et al., 1999; King, 2009; van Dalen et al., 2013). They have basically the same form in all areas. This means that the fast conversion tools based on conversion frames we have discussed may equally well be applied in the analysis of many other spectroscopic methods.

## Conclusions

By a ready-made panel-based conversion of ε′ to ε″ data the l.f. resolution of dielectric spectra of colloidal systems can be improved. The crux bringing about this improvement is that ε′ data will never contain any contribution of the ohmic conduction current, which is in phase with the applied a.c. voltage, whereas the current from the real or ε′ part is out phase.

A high resolution is also an issue when several relaxation processes occur in near unison. Simple avenues are proposed to enhance the resolution of nearby colloidal peaks. In addition to the use of 2 or 3 term differences of ε′ and ε″, a new way of modeling is advocated. The methods designed furthermore facilitate, by virtue of their resolving power, the proper correction of the data of colloids for electrode polarization.

## Author contributions

The author devised and tested the new methods described in the work. He contrived its intellectual content. He approves the final version. He is accountable for all aspects of it in ensuring that questions related to the accuracy or integrity are appropriately investigated and resolved.

### Conflict of interest statement

The author declares that the research was conducted in the absence of any commercial or financial relationships that could be construed as a potential conflict of interest.

## Footnotes

^1^Although *dε*′∕*dlnω* is a negative quantity, because ε′ drops with ω, we found it more convenient to plot and treat it below as a positive quantity. So in most cases we will actually deal with the absolute value of d ε′∕*dlnω*.

^2^We may envisage the use of fractional polynomials in iω as a modeling of the dielectric response with a parallel circuit of several Warburg impedances. By combining such polynomials in a ratio, we only need sums with a limited number of terms.

^3^This means that under these circumstances the theoretical model function for *dε*′/*dlnω* has to be calculated via a logarithmic difference with an interval h → 1, say of h = 10^0.05^ or less.

## Abbreviations

cos(π*D*_*l*_/2): cos-operator
cot(π*D*_*l*_/2): cot-operator
sin(π*D*_*l*_/2): sin-operator
tan(π*D*_*l*_/2): tan-operator
d: electrode distance
d_*l*_: Debye length
D: normal derivative
D_*l*_: logarithmic derivative
E: normal shift
E_*l*_: logarithmic shift
f(τ): distribution function
g(lnτ) = τf(τ): logarithmic distribution function
h: logarithmic spacing
i: imaginary unit
m^*^(ω): complex dielectric modulus
z^*^(ω): complex impedance
β^*^(ω): complex dipolar coefficient
ε^*^(ω): complex permittivity
ε_*a*_(ω): magnitude or absolute value
$\varepsilon_{cs}^{*}$ (ω): measured complex cell permittivity of suspension
ε_*dl*_: real permittivity of double layer
$\varepsilon_{e}^{*}$(ω): complex permittivity electrolyte
$\varepsilon_{ep}^{*}$ (ω): complex permittivity electrode polarization
ε${}_{s}^{*}$(ω): true permittivity of suspension
ε_*w*_: real permittivity of water
ε′: real part of permittivity
ε″: imaginary part of permittivity which may include conduction losses
$\varepsilon_{cf-rf}^{\prime \prime }$: ε″ free of conduction via ratio of fractional sums
ε″_*kk*_ = ε″_*cf*_: ε″ free of conduction via KK
γ: ohmic conductivity
γ_*e*_: ohmic conductivity of electrolyte
κ′(x): Debye's real kernel
κ″(x): Debye's imaginary kernel
τ: relaxation time
τ_*e*_: (ohmic) relaxation time electrolyte
*dε*′∕*dlnω*: logarithmic derivative of ε′
*dε*″∕*dlnω*: logarithmic derivative of ε″
Δε′(ω): logarithmic difference of ε′
$\Delta \varepsilon_{fc}^{\prime \prime }$(ω): asymmetric conduction free ε″ difference
Δε″_*cf*_(ω): symmetric conduction free ε″ difference.
